# COBRA-Seq: Sensitive and Quantitative Methylome Profiling

**DOI:** 10.3390/genes6041140

**Published:** 2015-10-23

**Authors:** Hilal Varinli, Aaron L. Statham, Susan J. Clark, Peter L. Molloy, Jason P. Ross

**Affiliations:** 1 CSIRO Food and Nutrition Flagship, North Ryde, New South Wales 1670, Australia; E-Mails: peter.molloy@csiro.au (P.L.M.); jason.ross@csiro.au (J.P.R.); 2 Genomics and Epigenetics Division, Garvan Institute of Medical Research, Darlinghurst, New South Wales 2010, Australia; E-Mails: a.statham@garvan.org.au (A.L.S.); s.clark@garvan.org.au (S.J.C.); 3 Department of Biological Sciences, Macquarie University, North Ryde, New South Wales 2109, Australia; 4 Vincent’s Clinical School, Faculty of Medicine, UNSW, New South Wales 2010, Australia

**Keywords:** COBRA, DNA methylation, reduced representation, non CpG, non-model organism, restriction enzymes, next generation sequencing, enhancer, CHH

## Abstract

Combined Bisulfite Restriction Analysis (COBRA) quantifies DNA methylation at a specific locus. It does so via digestion of PCR amplicons produced from bisulfite-treated DNA, using a restriction enzyme that contains a cytosine within its recognition sequence, such as *TaqI*. Here, we introduce COBRA-seq, a genome wide reduced methylome method that requires minimal DNA input (0.1–1.0 μg) and can either use PCR or linear amplification to amplify the sequencing library. Variants of COBRA-seq can be used to explore CpG-depleted as well as CpG-rich regions in vertebrate DNA. The choice of enzyme influences enrichment for specific genomic features, such as CpG-rich promoters and CpG islands, or enrichment for less CpG dense regions such as enhancers. COBRA-seq coupled with linear amplification has the additional advantage of reduced PCR bias by producing full length fragments at high abundance. Unlike other reduced representative methylome methods, COBRA-seq has great flexibility in the choice of enzyme and can be multiplexed and tuned, to reduce sequencing costs and to interrogate different numbers of sites. Moreover, COBRA-seq is applicable to non-model organisms without the reference genome and compatible with the investigation of non-CpG methylation by using restriction enzymes containing CpA, CpT, and CpC in their recognition site.

## 1. Introduction

Extensive effort has been devoted to mapping the methylome of various human tissues [1,2,3]. Genome-wide methylation is also widely studied in plants, *Arabidopsis thaliana* [4,5,6,7,8,9], *Oryza sativa* (rice) [5,8,10], and several model species ranging from fungi [8,11,12,13], *Drosophila melanogaster* (fruit fly) [14,15], *Apis mellifera* (honey bee) [16], *Danio rerio* (zebrafish) [5,17,18] and *Mus musculus* (mouse) [5,19,20]. Methylome analysis is also beginning to be applied to livestock species such as sheep [21]. Moreover, there is a growing interest in understanding the molecular bases of epigenetic inheritance in non-model organisms [22], including organisms of ecological and economic importance (*i.e.*, root-knot nematodes [23]).

The gold standard technique for quantifying methylomes is whole-genome bisulfite sequencing (WGBS) [1,6,24]. Bisulfite treatment converts cytosines to uracil while 5-methylcytosines (5mC) are preserved [25,26]. Thus, in subsequent sequencing of bisulfite-treated DNA the 5mC are read as cytosines, and unmethylated cytosines as thymines at a single base resolution. Although WGBS gives nucleotide-base resolution across the entire genome, in humans, only about 20% of the CpGs are reported as being dynamic (varying by more than 30%) among various human tissues [27]. WGBS is currently too expensive to generate large-scale data on multiple cell types or large sample sets. It is even more challenging in systems biology research that involves longitudinal sampling of multiple tissue and cell types in large cohorts, particularly in the case of biomarker discovery.

While methylation can occur in a variety of sequence contexts in plants and other organisms, vertebrate genomes are methylated predominantly at the dinucleotide CpG [28]. Empirically, it has been shown that 65% of 100 bp WGBS reads of mammalian DNA contain no CpG sites [27]. Consequently, much of the sequencing bandwidth of WGBS is wasted. Further, methylomes with low levels of 5mC, such as those from insect taxa [29] demonstrate an even more pronounced waste of sequencing bandwidth. For example, less than 1% of the CpGs are methylated in the honeybee genome [16]. There is a growing interest in profiling only a representative subset of cytosines across the genome to limit cost, and increase cohort size, regardless of the choice of organism.

A wide number of microarray and sequence-based DNA methylation detection and analysis methods have been developed that target portions of the genome and reduce genomic complexity [30,31,32,33,34,35,36,37,38,39]. Each method targets a different subset of the methylome, revealing methylation profiles across a different distribution of genomic features (*i.e.*, CpG rich and/or poor regions) [40]. There are three main approaches that exploit next-generation sequencing to derive methylome data: bisulfite conversion, affinity enrichment and enzymatic restriction using methylation sensitive endonucleases.

The traditional Combined Bisulfite Restriction Analysis (COBRA) method quantifies DNA methylation in a specific gene region by PCR amplification from bisulfite-treated DNA. This is followed by digestion of the PCR amplicon with a restriction enzyme such as *TaqI* (5'-T/CGA-3') that contains a CpG site in its recognition sequence. The methylation status of a particular CpG site within an enzyme recognition site can then be determined. The proportions of cut *vs*. uncut DNA can be used to determine the level of methylation [41].

Here, we have optimized the original COBRA method for high-throughput genome sequencing platforms and refer to the method as COBRA-seq. COBRA-seq enriches methylated DNA fractions by digesting the genomic DNA with restriction enzymes recognizing potential methylation sites after bisulfite conversion. The genomic complexity is further reduced by removing DNA fragments without the enzyme recognition site using streptavidin coated magnetic beads. Therefore, COBRA-seq provides single base pair resolution data within multiple regions of interest containing methylated sites. COBRA-seq is compatible with various restriction enzymes allowing the user to explore 5mC in any sequence context (CpA, CpT, CpC as well as CpG).

We describe two versions of COBRA-seq: First, Genome-Wide COBRA (GW-COBRA) that uses PCR to exponentially amplify the sequencing library fragments after linker ligation. Second, Linear Amplification COBRA (LA-COBRA) that relies on T7 RNA polymerase-mediated transcription yielding many RNA transcripts of the library fragments generated in a linear manner which are subsequently converted to cDNA. The COBRA-seq library protocol is largely adapted to provide robust coverage of the human genome, however, all of the steps are applicable to both model and non-model organisms.

We prepared GW-COBRA and LA-COBRA sequencing libraries from the colon carcinoma (HCT116) cell line and analyzed the methylome with 100 bp single-end sequencing using Illumina HiSeq2000 chemistry. We also compared, *in silico*, the quantitative, qualitative and genome coverage specifications of COBRA-seq with other common DNA methylome technologies.

## 2. Materials and Methods

### 2.1. Cell Culture and Genomic DNA Isolation

HCT116 colon cancer cells were cultured in McCoy’s 5A media (Life Technologies, cat#16600-082, Carlsbad, CA, USA) supplemented with 10% fetal bovine serum (Life Technologies, cat#10099-141). Genomic DNA was isolated using a Gentra Puregene Cell Kit (Qiagen, cat#158745, Redwood City, CA, USA) as per manufacturer’s instructions. Purified genomic DNA was quantified with a Nanodrop ND-1000 (Thermo Scientific, Carlsbad, CA, USA).

### 2.2. Annealing Oligonucleotides to Construct COBRA-Seq Adapters

A final concentration of 50 μM annealed adapter stocks were prepared using the corresponding oligonucleotide pairs described below in 1× Quick Ligation™ Reaction Buffer (NEB, supplied in cat#M2200S, Ipswich, MA, USA). The reactions were heated to 95 °C for 5 min then cooled down gradually as follows: 72 °C for 5 min, 60 °C for 5 min, 50 °C for 3 min, 40 °C for 3 min, 30 °C for 3 min, 20 °C for 3 min, 10 °C for 3 min and 4 °C for forever. The annealed adapter stocks were stored in −20 °C.

*GW-COBRA and LA-COBRA Adapter 2 (A2)*. A2, has a T-3' overhang, composed of A2-LowerStrand and A2-UpperStrand oligonucleotide pair provided in Table 1, were modified from the original TruSeq Illumina Y-shaped Adapter 2 oligonucleotides. All the cytosines were changed to 5mCs in the lower strand (A2-LowerStrand), hence the sequence were maintained post-bisulfite treatment. On the upper strand most cytosines in the flanking end were changed to 5mCs. Thus after bisulfite treatment, sequences in the stem of the adapters are no longer complementary. After bisulfite treatment the sequence on the fragment ending with A2-LowerStrand oligonucleotide sequence is appropriate for next-generation sequencing flow cell amplification.

**Table 1:** Details of oligos used in constructing GW-COBRA and LA-COBRA methylome libraries. The less common sequence abbreviations are: 5 (5mC), b (5' two biotin groups) and p (5' phosphorylation).

Primer Name	Primer Sequence (5'–3')*	Purification Method
**A2-UpperStrand**	TGT 5A5 5G5 TGG T5A T5C GCT GCT CTT CCG ATC T	PAGE and HPLC
**A2-LowerStrand**	pGAT 5GG AAG AG5 T5G TAT G55 GT5 TT5 TG5 TTG	PAGE and HPLC
**GW-A1-UpperStrand**	CTA CAC TCT TTC CCT ACA CGA CGC TCT TCC GAT CT	PAGE and HPLC
**GW-A1-LowerStrand**	CGA GAT CGG AAG AGC GTC GTG TAG GGA AAG AGT GTA G	PAGE and HPLC
**LA-A1+P5-UpperStrand**	AAT GAT ACG GCG ACC ACC GAG ATC TAC ACT CTT TCC CTA CAC GAC GCT CTT CCG ATC T	PAGE and HPLC
**LA-A1+P5-LowerStrand**	CGA GAT CGG AAG AGC GTC GTG TAG GGA AAG AGT GTA G AT CTC GGT GGT CGC CGT ATC ATT	PAGE and HPLC
**GW-A2-FwdP**	CAA GCA GAA GAC GGC ATA CGA GCT CTT CCG ATC T	PAGE
**COBRA-A2-RevP**	**b**TGT CAC CGC TGG TCA TCT GTT GTT T	HPLC
**LA-A2+T7-FwdP**	GAA TTT AAT ACG ACT CAC TAT AGG GAC AAG CAG AAG ACG GCA TAC GAG C	PAGE
**FlowCell-FwdP**	AAT GAT ACG GCG ACC ACC GAG ATC TAC ACT CTT TCC CTA CAC GAC GCT CTT CCG ATC T	HPLC
**FlowCell-RevP**	CAA GCA GAA GAC GGC ATA CGA GCT CTT CCG ATC T	HPLC
**LADS P5**	AAT GAT ACG GCG ACC ACC GA	HPLC
**LADS P7**	CAA GCA GAA GAC GGC ATA CGA	HPLC

*GW-COBRA Adapter 1 (GW-A1)*. GW-A1, has a 5'-CG overhang, composed of GW-A1-UpperStrand and GW-A1-LowerStrand oligos in Table 1.

*LA-COBRA Adapter-1 (LA-A1)*. LA-A1, composed of LA-A1+P5-UpperStrand and LA-A1+P5-LowerStrand oligos in Table 1, also has a 5'-CG overhang and contains an addition of the P5 primer region that allowed reverse transcription and cDNA synthesis. Both GW-A1 and LA-A1 contained the appropriate end for flow cell amplification when paired with A2.

### 2.3. Library Construction and Bisulfite Treatment

*Fragment Preparation*. The genomic DNA was resuspended in 300 μL low TE (10 mM Tris, 0.1 mM EDTA, pH 7.5) to a final concentration of 16.66 ng/μL and fragmented using a Bioruptor UCD-200 sonicator (Diagenode) at a power setting of “high” for sets of 30 cycles of 15 s on/off with 15 min intervals on ice in between each set. The fragmented DNA, 100–500 bp, was concentrated using ethanol precipitation. Each replicate containing 2 μg DNA was end repaired using the End-It™ DNA End-Repair Kit (Epicentre Biotechnologies, cat#ER0720, Madison, WI, USA), repurified with the standard phenol:chloroform: isoamyl alcohol extraction, A-tailed using Klenow Exo- (NEB, cat#E6053S) as per manufacturer’s protocol then the reaction was inactivated at 75 °C for 20 min.

*Ligation of A2*. Ligation was performed using a Quick Ligation™ kit (NEB, cat#M2200S) in the presence of a 10-fold excess of A2 (310 pmol for 2 μg of genomic library with average fragment size of 200 bp) as per the manufacturer’s instructions. The DNA was cleaned up with a QiaQUICK PCR purification kit (Qiagen, cat#28104), eluted in 84 μL of elution buffer (EB).

*Bisulfite Conversion*. The bisulfite treatment of ligated genomic DNA was carried out with the EZ DNA Methylation Kit (Zymo Research, cat# D5001, Irvine, CA, USA) following the manufacturer’s protocol with minor modifications. The bisulfite conversion reaction was incubated at 99 °C 5 min, 60 °C 25 min, 99 °C 5 min, 60 °C 85 min, 99 °C 5 min, 60 °C 175 min and 22 °C 5 min. Yeast tRNA were added to low levels of genomic DNA (<0.5 μg) to minimize sample loss.

*Limited PCR Amplification*. Single-stranded bisulfite DNAs were used as PCR templates at a final concentration of 1 ng/μL in 25 μL using GoTaq colorless master mix (Promega, cat#M7133, Madison, WI, USA) [25]. The reverse primer contained 5' double biotin (COBRA-A2-RevP in Table 1). In addition to GW-COBRA forward primer shown as GW-A2-FwdP in Table 1, LA-COBRA forward primer (LA-A2+T7-FwdP in Table 1) had an overhang of T7 promoter region that allowed linear amplification of the library fragments via *in vitro* transcription in the later steps. The temperature cycles for the PCR were: 98 °C 3 min; 98 °C for 15 s, 56 °C for 30 s and 72 °C for 1 min, for 6 cycles; a final extension of 72 °C for 2 min. A Wizard SV PCR Clean Up System (Promega, cat#A9281) was used to remove the enzymes and excess primers as per the manufacturer’s instructions.

*Restriction Digestion and Enrichment of Methylated DNA Fragments*. PCR amplified and purified 2 μg library material was then digested overnight with 100 U of *TaqI* (NEB, cat#R0149S) in NEB buffer 4 in a final volume of 100 μL at 65 °C. After *TaqI* digest, there are three main fragment types in the libraries; uncut biotinylated fragments (no internal *TaqI* site containing 5' double biotin), the cut fragments containing 5' double biotin and the other part of the cut fragments which are non-biotinylated. Dynabeads^®^ M-280 Streptavidin beads (Life Technologies, cat#11205D) were used to capture the biotinylated fragments as per manufacturer’s instructions, hence enriching the non-biotinylated fragments in the eluate [42]. The eluate was ran through QIAquick PCR purification column (Qiagen, cat#28104) and resuspended in water for the following ligation step. The biotinylated fragments were released from the beads with an incubation step for 15 min in 30 mM d-biotin (Sigma, cat#47868, Saint Louis, MO, USA) then heating to 80 °C for 15 min. A similar approach was used to release biotinylated proteins previously [43]. We compared the non-biotinylated fragment and biotinylated fragments on an agarose gel for QC.

*Ligation of GW-A1 or LA-A1 (5'-CG Overhang)*. The eluate containing 1 μg non-biotinylated, cut fragments were ligated to A1. The ligation was performed using a Quick Ligation™ (NEB, supplied in cat#M2200S) in the presence of 2-fold excess of GW-A1 or LA-A1 (16 pmol for 1 μg of genomic library with average fragment size of 200 bp), as per the manufacturer’s instructions. The excess A1 and fragments less than 100 bp were removed with Agencourt AMPure XP Bead system (Beckman Coulter, cat# A63880, Brea, CA, USA) using the TruSeq DNA sample preparation guide with minor modifications: a ratio of 125 μL of well mixed beads with 135 μL of sample.

*GW-COBRA Library PCR Amplification*. Library fragments containing A1 and A2 were PCR amplified, with FlowCell-FwdP and FlowCell-RevP primers (Table 1), in a final concentration of 1 ng/μL in 25 μL using GoTaq colorless master mix (Promega, cat#M7133). The temperature cycles for the PCR were: 98 °C 3 min; 98 °C for 15 s, 65 °C for 30 s and 72 °C for 1 min, for 7 cycles; a final extension of 72 °C for 2 min.

*LA-COBRA Library Linear Amplification*. Ligated library fragments were *in vitro* transcribed to RNA using T7 RNA Polymerase Kit (NEB, cat#E2040S) at 37 °C for 16 h, then cleaned up with RNeasy MinElute Clean up kit (Qiagen, cat# 74204) and quantified using Quant-iT RNA assay (Life Technologies, cat#Q-33140) respectively as per manufacturers’ protocol. The cDNA library was constructed using 600 ng RNA using the SuperscriptIII first-strand synthesis kit (Life Technologies, cat#18080-051) and the LADS P5 primer. Following treatment with RNase H, cDNA was made double stranded using the Klenow fragment of DNA Polymerase 1 and the P7 primer as described in [44].

*Library Clean-up*. Agencourt AMPure XP Beads (Beckman Coulter, cat# A63880) at a ratio of 125 μL of well mixed beads with 135 μL of sample were used to remove short fragments from both GW-COBRA and LA-COBRA libraries. Finally, the size distribution was visualized using Agilent DNA 1000 Assay in 2100 Bioanalyzer (Agilent Technologies, Los Angeles, CA, USA) using the manufacturer’s protocol. Throughout the COBRA-seq library construction, appropriate products were ran on either 3% low range ultra-melting agarose gel (Bio-rad, cat# 161-3107, CA, USA) or a 4%–20% Criterion precast polyacrylamide TBE gel (Bio-rad, cat#345-0059) and stained with SYBR gold (Life Technologies, cat#S-11494).

*Library Sequencing*. GW- and LA-COBRA libraries of HCT116 DNA were sequenced with the 100 bp single-end Illumina HiSeq 2000 technology in a single lane each at the Australian Genome Research Facility. The COBRA library sequencing results are deposited in the CSIRO Data Access Portal which are accessible with the manuscript title search [45].

### 2.4. Bioinformatics and Statistics

*Alignment*. FASTQ reads were inspected for adaptor contamination and a set identified. This set, with the specification of at least an 8 base overlap, were subsequently removed using the fuzzy matching functionality of cutadapt [46]. Reads were also quality trimmed and length filtered by cutadapt using a quality setting of “-q 8” and minimal read length of 40 bases. The cutadapt processed reads were aligned with bwa-meth [47], a bisulfite-treated DNA tuned wrapper for the BWA-MEM aligner [48].

*Clean up and Count Statistics*. The alignments were further processed using a Python script. This script examined each alignment and excluded any alignment (by setting the alignment as unmapped; bit flag 4) if the alignment was identified as being a secondary alignment, did not have the expected three remaining bases of the *TaqI* restriction site, or did not align to an *in silico* identified *TaqI* site. Recognising the error-prone nature of sequencing, the three base *TaqI* site match was relaxed to a Levenshtein distance of 1. This fuzzy matching allowed a one base mismatch between the first three bases of a forward strand read and a “CGA” trimer, or the last three bases of a reverse strand read and the “TCG” trimer sequence. Forward alignments that started, or reverse alignemnts that ended on the exact genome coordinate of an *in silico* determined *TaqI* cut site were kept and tallied by *TaqI* site. A record of the counts per *TaqI* site were exported as a bedGraph file. The file was used for visualisation in the IGV genome browser and was also imported into R for further analysis. The read cleaning procedure is provided in Figure S1.

*In silico Analyses*. Mapping of CpG sites, *in silico* bisulfite DNA treatment and *in silico* restriction enzyme digests on both strands of bisulfite-converted DNA were written in R using the functionality of the Bioconductor Biostrings and GenomicRanges libraries and the BSgenome.Hsapiens.UCSC.hg19 genome build library. Annotations were from the TxDb.Hsapiens.UCSC.hg19.knownGene library or downloaded from the UCSC web server via rtracklayer. Selection was further restricted to fragments greater than 70 bp as small fragments will be selected against through the library preparation process.

CpG island locations used were those in the “CpG Islands” UCSC table. CpG Shores were defined as the area flanking 2 kb of an island. There were 2,089,538 and 2,089,538 CpGs located in CpG Islands and shores respectively. The remaining CpGs were classified as CpG Ocean (24,105,864 CpGs). CpGs within 4 kb distance to transcription start sites were determined to be located in promoters (3,619,885 CpGs). The gene body CpGs was defined as those in the area between gene start and end coordinates (12,121,165 CpGs). Intergenic CpGs are those CpGs not within the genebody or TSS category (12,476,398 CpGs). Enhancer sites (205,740 CpGs) were defined as those within the start and end coordinates of FANTOM5 permissive enhancers [49].

*Comparison to Other Data*. The HCT-116 450K array was processed and beta values called using the R minfi library. All bioinformatics analysis scripts are deposited to the repository, along with a tutorial outlining their use [50].

## 3. Results and Discussion

### 3.1. COBRA-Seq Library Construction

**Figure 1:**
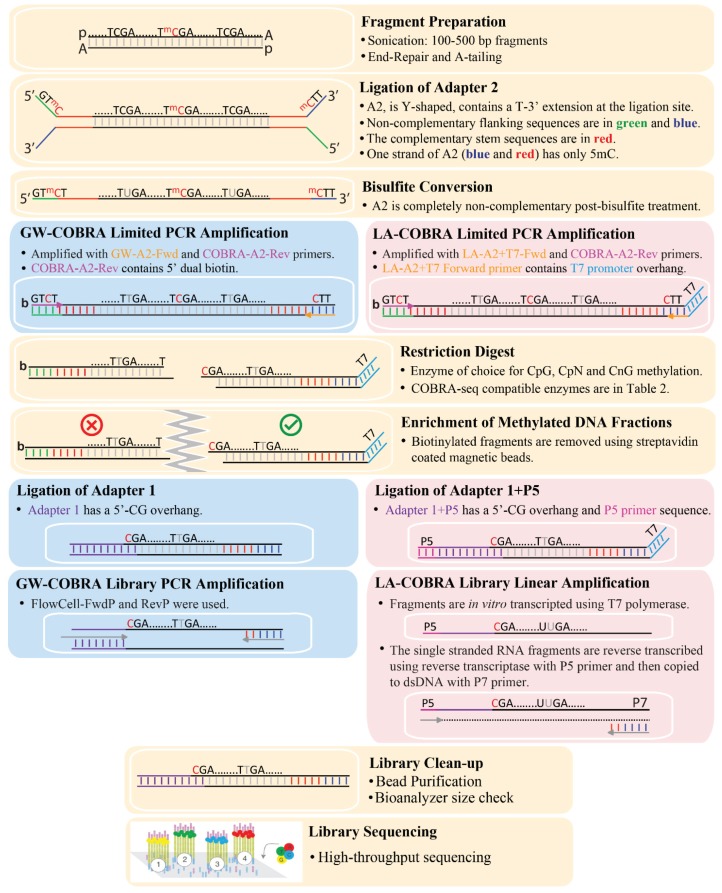
Flowchart of key steps in constructing COBRA-seq libraries with minor steps omitted for clarity. The common steps in both methods are shown on light yellow background. The GW-COBRA and LA-COBRA specific steps are on light blue and pink backgrounds respectively. The less common sequence abbreviations are: ^m^C (5mC), b (two biotin groups sequentially placed on the 5'-end) and p (5' phosphorylation).

**Figure 2:**
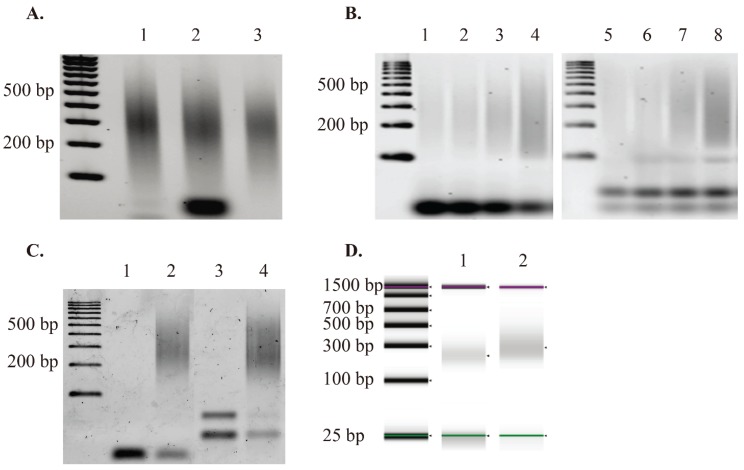
GW-COBRA and LA-COBRA library construction results. (**A**) Sonicated genomic DNA isolated from HCT116 cell line (lane 1), 50 ng of Adapter-2 ligated library material (lane 2). Column purified Adapter-2 ligated library material (lane 3); (**B**) PCR amplification test of bisulfite treated library materials. Lane 1, 2, 3 and 4 are produced by 6, 7, 8 and 9 cycles of PCR using GW-A2-FwdP and COBRA-A2-RevP primers from Table 1 with GW-COBRA library material as the template respectively. Lane 5, 6, 7 and 8 are produced by 6, 7, 8 and 9 cycles of PCR using LA-A2+T7-FwdP and COBRA-A2-RevP primers from Table 1 with LA-COBRA library material as the template, respectively; (**C**) Final library products: GW-COBRA and LA-COBRA methylome libraries of HCT116 DNA amplified using flowcell or LADS primer pairs respectively (Lane 2 and 4). Lane 1 and 3 are negative controls for GW-COBRA and LA-COBRA libraries, respectively; (**D**) Bioanalyzer results of GW-COBRA and LA-COBRA methylome sequencing libraries, respectively, prepared from HCT116 cell line DNA. The final library fragments ranged between 150–500 bp with an average size of 257 and 360 bp for GW-COBRA and LA-COBRA, respectively.

The procedure for preparation of COBRA-seq libraries is outlined in Figure 1, described in detail in the Materials and Methods and Supplementary Materials and Methods. The oligonucleotides used are shown in Table 1 and Figure S2. We applied the method to prepare COBRA-seq libraries from HCT116 cell line DNA (Figure 2). Briefly, genomic DNA was fragmented and end-repaired as for normal genomic library construction. Sonicated HCT116 genomic DNA ranged between 150–500 bp (Figure 2A). After bisulfite treatment of the ligated DNA, the libraries were subjected to a minimal number of PCR cycles in order to replace uracils in the original DNA with thymines (some restriction enzymes do not cut uracil-containing DNA efficiently). The A2 reverse primer, used in the PCR step, contains two biotin groups sequentially placed on the 5' end. While the ligation of Adapter-2 did not generate a visual shift in the sonicated library size, ligation was verified by amplification with the GW-A2 Fwd and COBRA-A2-RevP or LA-A2+T7 Fwd and COBRA-A2-RevP primers pairs (Figure 2B).

For the pilot study, we chose *TaqI* (5'-T/CGA-3') for complexity reduction as it has historically widespread usage in the traditional COBRA method. *TaqI* and a number of other enzymes contain CG within their recognition sequences and are therefore suitable for use with mammalian DNA that is principally methylated at CpG sites. COBRA-seq with *TaqI* covers nearly 16% of the CpG sites in the human genome. After digestion of the dual biotin-labelled material with *TaqI*, cut fragments will have one of the pair tagged with a dual biotin while the other cleaved fragment will not; all uncut molecules will contain a dual biotin tag at one end. Streptavidin-coated magnetic beads are then used to remove biotin-labelled material, the uncut molecules as well as one end of the cut fragments. The remaining fragments, containing a –CG overhang, are ligated with Illumina Adapter 1 (GW-A1 and LA-A1) linkers, modified with a –CG extension. Therefore, COBRA-seq libraries contain only methylated CpG sites and give relative methylation level based on read counts.

For GW-COBRA, the ligated products are amplified by PCR and quantified prior to sequencing. In LA-COBRA, the Adapter 2 (A2) is modified to include a phage T7 RNA polymerase promoter site (right hand side of Figure 1). After ligation of LA-A1, T7 RNA polymerase is used to make RNA copies of the ligation products. Reverse transcriptase is used in combination with the P5 primer to copy these into cDNA. After RNaseH digestion, copies are made double-stranded using the P7 primer and the Klenow fragment of DNA polymerase 1. LA-COBRA produces full length fragments at high abundance via *in vitro* transcription and importantly removes PCR bias, which is a common side effect of amplifying bisulfite-treated DNA [51]. The final GW-COBRA and LA-COBRA library products are checked using Illumina Flowcell primer pairs or Linear Amplification for Deep Sequencing (LADS) primer pairs published in [44] (Figure 2C).

The final library fragments is ranged between 150–500 bp with an average size of 257 and 360 bp in GW-COBRA and LA-COBRA, respectively (Figure 2D). The amount of library material is recovered for each protocol averaged 450 ng for LA-COBRA and 50 ng for the PCR based GW-COBRA.

During COBRA-seq method development, we also examined the effect of limited amounts of starting material. Both GW-COBRA and LA-COBRA libraries were prepared with as little as 0.1 μg of genomic DNA of HCT116 cells which did not restrict the success of the library preparation protocol. The size distribution of the COBRA-seq library fragments was the same when the starting material was either 0.1 or 1.0 μg (Figure S3).

### 3.2. Number of GW-COBRA and LA-COBRA Reads and Mapping to Genome

Libraries were sequenced in a single lane each, on a 100 bp single-end in Illumina HiSeq2000 run, generating 115,097,029 and 142,245,797 million reads for GW-COBRA and LA-COBRA, respectively, with 83.1% and 84.1% of the reads mapped uniquely (Table S1). The bisulfite conversion rate was near complete (99.4%, FastQC) (Table S1). Consistent with T7 RNA polymerase having a higher nucleotide misincorporation rate than *Taq* polymerase [52], we observed LA-COBRA reads contained more sequence errors but this did not interfere with the mapping; 92.34% of the LA-COBRA library reads were mappable compared with 92.53% for GW-COBRA (Table S1). The FASTQC summary results are provided in Figure S4. Median read coverage for *TaqI* sites with at least one read were 8 and 9 for GW-COBRA and LA-COBRA, respectively. Density plots of the CpG site coverage in the HCT-116 GW-COBRA and LA-COBRA libraries and public RRBS and WGBS libraries are provided in Figure S5.

**Figure 3:**
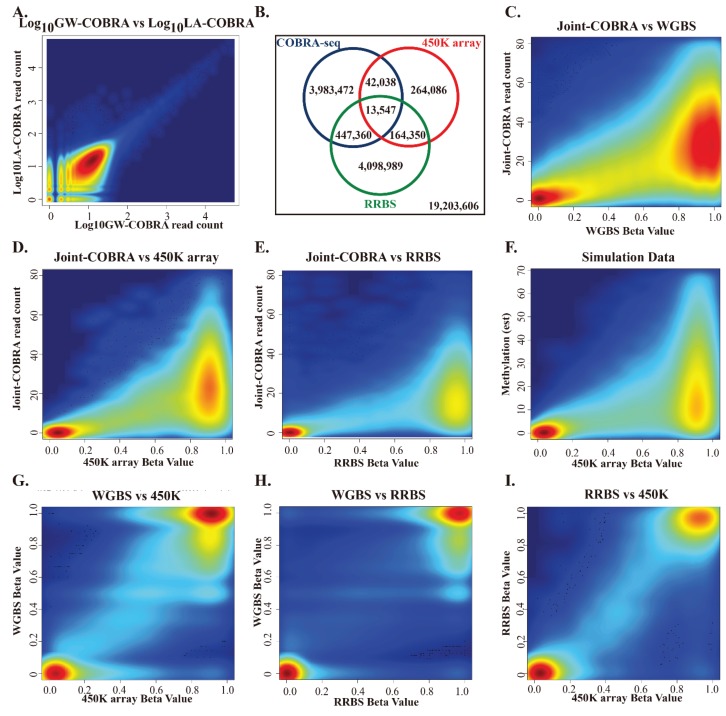
Empirical comparison of HCT116 COBRA-seq methylome with RRBS and 450K array data. (**A**) Comparison of the GW-COBRA and LA-COBRA log transformed read counts aligning to the same genome position (5,756,193 *TaqI* sites, R^2^ = 0.905); (**B**) Venn diagram comparing coverage of CpG sites by the 3 methods; (**C**) Combined methylome sequencing data of GW-COBRA and LA-COBRA (Joint-COBRA; median coverage of 17 reads per strand) compared with HCT116 cell line WGBS data (902,990 shared CpG sites, R^2^ = 0.384); (**D**) with HCT116 cell line 450K array data (31,988 shared CpG sites, R^2^ = 0.439) and (**E**) with HCT116 cell line RRBS data (95,766 shared CpG sites; median coverage of 82.14 reads per strand, R^2^ = 0.443); (**F**) Population mean (μ) coverage of 24.6 in M fraction only using simulated data; (**G**) Comparison of methylation scores of WGBS with 450K (463,300 shared CpG sites); (**H**) WGBS wish RRBS (978,735 shared CpG sites); (**I**) RRBS with 450K (46,374 shared CpG sites).

The empirical results demonstrate that representation of different DNA fragments was highly similar for LA-COBRA and GW-COBRA (Figure 3A and Figure S4). Frequencies of read counts at specific sites were highly concordant between the two methods (Figure 3A, R^2^ = 0.905). Because of this high concordance, we joined the datasets for comparisons with other methods (below).

### 3.3. Comparison of HCT116 COBRA-Seq with RRBS and 450K Array Data

We compared our HCT116 COBRA-seq methylome data with WGBS data on HCT-116 cells and data obtained from two widely used methods that also sample a proportion of the genome: Illumina Infinium HumanMethylation450 BeadChip arrays (450K arrays) [30] and Reduced Representation Bisulfite Sequencing (RRBS) [53]. Each method targets a different subset of the methylome, with some overlapping CpG sites (Figure 3B). On microarrays, a very large number of molecules bind to each array feature, giving an analogue estimation of the population mean. In particular, the 450K array is known for robustness and has been a popular choice due to its affordable price and high precision. The 450K beta (proportion of methylation) values can be considered as an accurate quantification across ~480,000 (1.7%) CpG sites. HCT116 450K methylation data is made available in our repository [50].

RRBS is an efficient high throughput methylome method that samples a population of restriction fragments isolated following digestion with the methylation-insensitive enzyme, *MspI*. This DNA population, encompassing 16.7% of CpG sites, is then bisulfite-treated and sequenced, and so provides reads of both methylated and unmethylated sequences. We used HCT116 cell line WGBS and RRBS methylation data (GSM1465024 and GSM919980) arising from published studies [54,55].

We also considered simulated data to provide an overview of the expected data distribution and concordance at different levels of sequence coverage with two types of methylome methods: one that samples both methylated (M) and unmethylated (U) fractions of the genome such as RRBS (M + U method in Figure S6, left panel) and methods that enrich for methylated (M) fractions like COBRA-seq (M only method in Figure S6, right panel).

We used empirical COBRA-seq over-dispersion estimates and 450K CpG site beta distribution data to inform the simulation. Across different depths of hypothetical sequencing coverage, 200,000 sample read counts were modelled, conditional upon the distribution of beta values from an HCT-116 450K array (Figure S6). For M + U methods, as read coverage increases, the ratio of methylated to unmethylated read counts will become more accurate and estimated beta will converge to the true beta. As beta is a ratio, at low coverage, under- or over-sampling of the M or U fraction will result in high imprecision in beta value estimation near the top (1.0) and bottom (0.0) of the beta value range. For the M only method, the read count will increase conditionally on both coverage and methylation rate, except where the CpG site is completely unmethylated; in this instance, increased coverage will not yield increased read counts. So, instead of the beta-binomial distribution of M + U methods, a zero-inflated count-based distribution will be observed, which is analogous to RNA-seq count data. CpG site state is not independent of other sites, so the distribution is not Poisson, but a family of Poisson distributions (negative binomial). Details of the modeling process and data are discussed in more detail in Supplementary Methods and Materials and Figure S6. In summary, we find that M + U methods have poor estimates at betas near 0 or 1 with low read coverage, but estimated beta rapidly forms a good correlation with true beta with increased read coverage. For M only methods, read coverage and methylation rate are confounded so estimates of partial or complete methylation are difficult. Instead, M only methods are best suited to situations where there are biological replicates. This allows modelling the within-group variance and the effects of library size, allowing coverage and methylation rate to be decoupled. The simulation suggests M only methods, like COBRA-seq, may have advantages over M + U methods in detecting lack of methylation between replicated experimental groups at low read coverage.

When considering read coverage requirements, our simulations suggest a 10 to 15-fold coverage with M + U methods is sufficient for reasonable beta estimates. An empirical comparison mapping 200,000 HCT-116 450K array beta values to WGBS beta values gives much the same estimate (Supplementary Figure S7). For M-only methods, we suggest a 10 to 15-fold coverage is sufficient and for extra sensitivity the addition of extra samples should be considered over additional read coverage.

We also wished to make an empirical comparison between COBRA-seq and other methods. For this, we plotted data for CpG sites shared between Joint-COBRA and WGBS data (902,990 shared CpG sites with at least 10-fold WGBS coverage), 450K array data (31,988 shared CpG sites) and RRBS data (95,766 shared CpG sites) (Figure 3C–E). The empirical data was a good fit with our simulated expectation (Figure 3D compared with 3F). Higher COBRA-seq read counts were associated with higher methylation rate (as determined by the comparison method). In particular, it should be noted that sites determined to be unmethylated by WGBS, RRBS or 450K array did not yield COBRA-seq reads, demonstrating the underlying principle of our method is sound.

**Figure 4:**
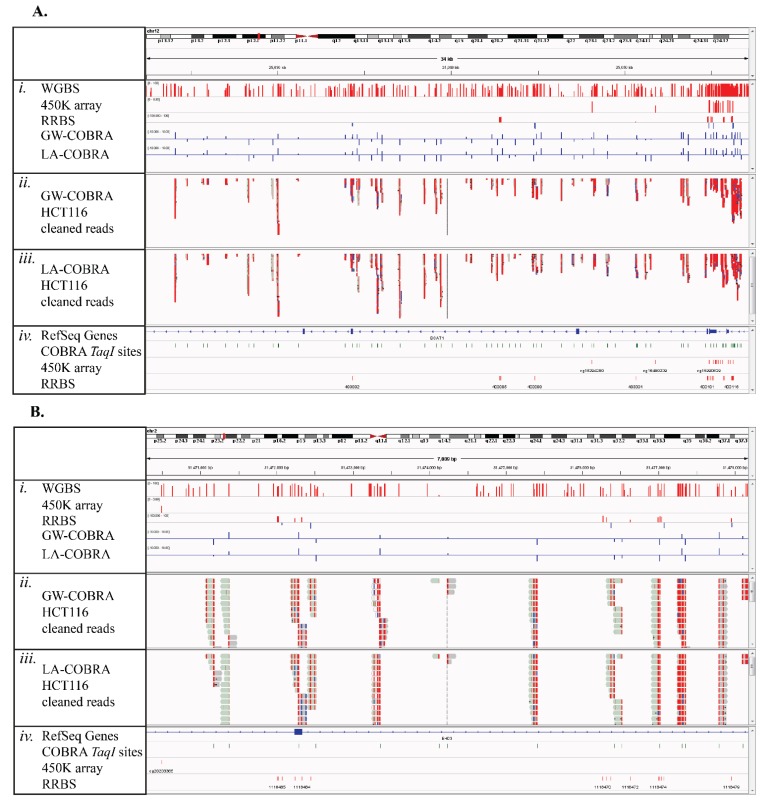
IGV screenshots on the selected colon cancer associated genes: (**A**) *BCAT1* and (**B**) *EHD3*. *i*. Methylation levels determined by WGBS, 450K arrays, RRBS GW-COBRA and LA-COBRA respectively; *ii*. Genome coverage and stacking of cleaned GW-COBRA reads; *iii*. Genome coverage and stacking of cleaned LA-COBRA reads; *iv*. Genomic locations of accessible COBRA *TaqI* sites, 450K array probes and accessible RRBS sites.

For a closer inspection across individual genome locations, we visualized the HCT116 cell line methylation profiles determined by GW-COBRA, LA-COBRA, WGBS, RRBS and 450K methods using the integrated genome viewer (IGV). Profiles of methylation across two genes, *BCAT1* and *EHD3*, which have been previously identified as biomarkers for colorectal cancer [42,56], are shown in Figure 4A,B. These regions are zoomed in to display a number of bases only in Figure S8. Additionally, more examples of the comparative methylation profiles across panel of genes (*SEPT9*, *MGMT*, *SLC6A15* and *FGF5*), highly methylated in colorectal cancer [57], are provided in Figure S9.

The methylation profiles generated by GW-COBRA and LA-COBRA have high concordance (Figure 4A,B; *ii*. and *iii*.), and also correlated highly with WGBS, RRBS and 450K array data (Figure 4A,B; *i*.) exemplified at the CpG rich promoter region of the *BCAT1* gene (Figure 4A). COBRA-seq provides a more even genomic distribution, compared to the bias towards CpG rich transcription start sites observed in 450K arrays and the RRBS method (Figure 4 and Figure S8; *iv*). The difference in locality of cutting between *TaqI* (COBRA-seq) and *MspI* (RRBS) can be explained by the GC-richness of the restriction site.

### 3.4. COBRA-Seq Features

As COBRA-seq fragments should start with the 3' end of the enzyme recognition site and should align to restriction sites in the genome, these factors can be used to clean up the quality of the sequencing data and alignments. The read cleaning procedure is visualized in IGV and reported in Figure S1 with a brief description of the procedure in the Materials and Methods. The cleaning procedure was conservative, keeping only reads that aligned to *in silico* predicted *TaqI* sites and requiring the reads started with *TaqI* site nucleotides, with a one base mismatch tolerance. We saw evidence that around half of the discarded reads were of high quality and had a *TaqI* site start (Table S2), but they aligned to *TaqI* sites not predicted in the reference genome. We also only considered methylation of the CpG site at the start of the read although it is possible to consider methylation at CpG sites within the read.

As COBRA-seq reads arise from bisulfite-treated DNA, it is possible to examine methylation at cytosines sites within a COBRA-seq read, however this has caveats. By design, reads depend of methylation of a cytosine within the enzyme recognition sequence. Therefore any observations at cytosines sites within reads are conditional upon methylation within the enzyme recognition sequence. While this confounds conventional methylation estimation, it is useful for examination of detailed methylation profiles, heterogeneity and of local co-methylation, which is a well-documented property of DNA methylation. Also, understanding the methylation status of cytosines within reads may be used qualitatively as evidence for determining the best sites for biomarker assay (primer) design.

One of the features of COBRA-seq is that a variety of enzymes can be used to assess 5mC in different contexts. Table S2 provides a selection of restriction enzymes that are suitable for employing COBRA-seq in different sampling depths and contexts. This table includes examples of 4 and 6-base cutter enzymes that cleave to leave a CG 5' overhang and are particularly useful for studies of CpG methylation (*AclI*, *BstBI*, *ClaI*, *HpyCH4IV* and *TaqI*). Enzymes containing CNG within their recognition sequence such as *HpyCH4III* and *Hpy188I* are potentially useful for studies of plant CpNpG methylation.

Some enzymes contain a cytosine at the end of their recognition site and hence can be used to monitor methylation independent of the nature of the neighboring nucleotide. For instance, *Sau3AI*, with the 5'-/GATC-3' recognition site, is a 4-base cutter. However, in the context of COBRA-seq, *Sau3AI* acts like a 5-base cutter as the recognition site can only be preserved if the 3' cytosine is methylated. In vertebrate DNA, this would typically be in a CpG context, whereas in plants, insects or any other genome, it can identify adjacent methylation in any context (CpN methylation). Similarly, *CviQI* and *EcoRI*, with the 5'-G/TAC-3' and 5'-G/AATTC-3' recognition sites respectively, are good choices for CpN methylation profiling in organisms such as insects. There are also enzymes that contains multiple CpG sites at their recognition sites such as *HinP1I* (5'-G/CGC-3') and *Hpy99I* (5'-CGACG/-3'). Their recognition sites can only be preserved if both of the CpGs are methylated therefore the number of sites observed in the actual sequencing results can be lower than the *in silico* digest estimations. These enzymes would be a good selection for discovery of highly methylated regions, or for co-methylation studies.

The number of potentially addressable methylation sites within a genome is dependent on the cutting frequency of the enzyme(s) used. Estimates of addressable CpG sites with COBRA-seq ranges from 1.4% to 16% of the sites in the human genome are provided in Table S3 and explained further in Materials and Methods. Among COBRA-seq compatible enzymes; *HpyCH4IV* reaches the highest proportion of CpGs (Tables S2 and S3). It is important to note that linkers for ligation to the restriction enzyme-cut ends must be modified as necessary to provide complementarity to the fragment overhangs. Fortunately a number of useful enzymes provide a CG-5' overhang (Table S2). COBRA-seq libraries can be multiplexed to increase the percentage of accessible sites, or alternatively tuned to reduce sequencing costs with the use of 6-base cutters instead of 4-base cutters to interrogate fewer sites. A typical 6-base cutter will have around 1/8 of the sites of a 4-base cutter. It is possible to prepare libraries using more than one enzyme, but we recommend the users perform separate digestions for each enzyme of choice before pooling and ligating to generate libraries as concurrent multi-enzyme digests will result in small fragment sizes which may cause loss of potential CpG sites during purification steps targeting removal of excess Adapters.

Additionally, for organisms without a reference genome, COBRA-seq is complementary for use with Restriction-site associated DNA sequencing (RADseq), a restriction enzyme-based method which reduces genome complexity across target genomes for SNP discovery [58]. It is possible to use the same restriction enzyme sites, RADseq for SNP discovery and COBRA-seq for methylation quantitation, thereby providing a convenient platform for allele-specific methylation discovery on non-model organisms.

**Figure 5:**
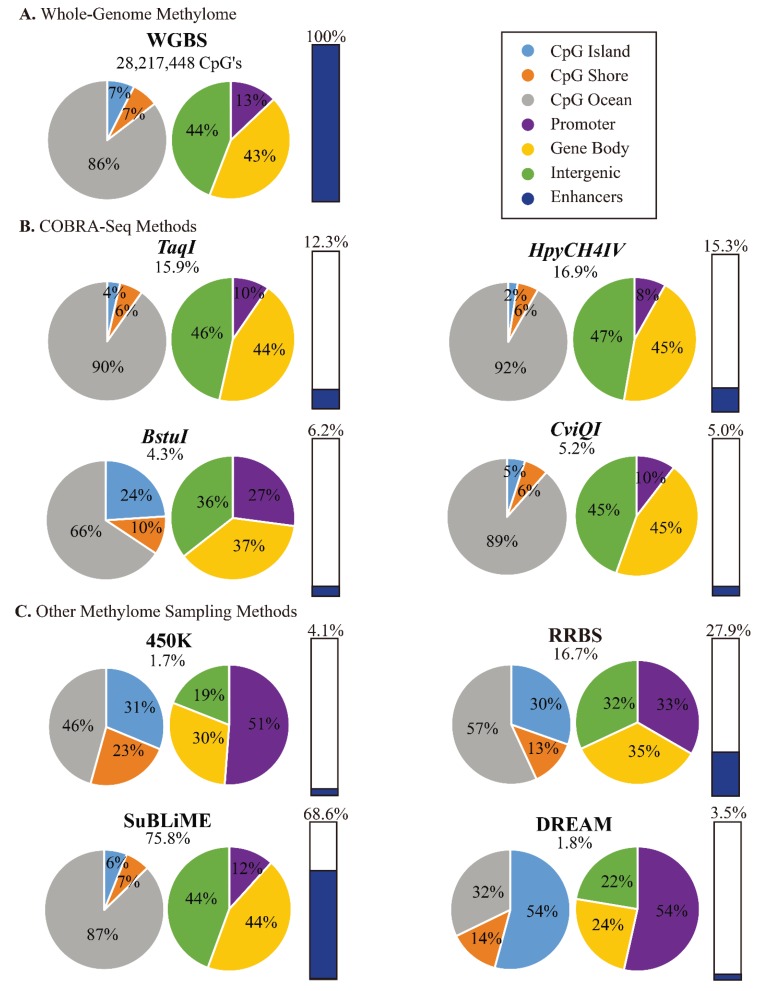
Comparison of CpG coverage of COBRA-seq with WGBS and commonly used methylome sampling methods by genomic regions, as pie charts or bar diagram for proportion of enhancer sites (WGBS covers a total of 205,740 CpGs in enhancers). (**A**) WGBS covers 28,217,448 CpG sites; (**B**) COBRA-seq with *TaqI*, *HpyCH4IV*, *BstuI* and CviQ*I* covers 15.9%, 16.9%, 4.3%, and 5.2% of the total CpG sites respectively; **C.** 450K arrays, RRBS, SuBLiME and DREAM cover 1.7%, 16.7%, 75.8%, and 1.8% of the total CpG sites respectively.

The COBRA-seq variation utilizing *TaqI* (5'-T/CGA-3') examined here has limited genomic bias due to the recognition site GC content and this in turn, yields relatively unbiased distribution across genomic annotations in human DNA: 4% and 6% coverage of CpG islands and shores respectively (Figure 5B and Table S3). It covers approximately 11.9% of genome-wide CpG sites within promoters and also similar coverage for features with fewer CpGs, such as enhancers (12.3%) and DNaseI hypersensitive (12.9%) sites (Figure 5 and Table S3).

In the COBRA-seq protocol described here we have used the standard protocol of ligation of adapters to sheared double-stranded DNA. However, it is also possible, and may be advantageous, to adapt library preparation methods that used single-stranded bisulfite-treated DNA as a starting point, e.g., Swift Accel-NGS-Methyl-Seq or Illumina EpiGnome/Tru-seq Methylation libraries. This could be particularly the case for DNA such as isolated from formalin-fixed tissue samples where DNA may be damaged or isolated single-stranded. Pre-treatment to repair damaged DNA (Illumina Restoration kit, Illumina, San Diego, CA, USA) may also improve the quality of such libraries.

### 3.5. Comparison of COBRA-Seq Features with Other Methods of Methylome Sampling

A range of different technologies for studying DNA methylation on a genome scale have become available, each with inherent differences in resolution, coverage and biases [59,60]. COBRA-seq quality control, read alignment and data visualization are the broadly the same as any WGBS or RRBS protocol [61]. However, methylation scoring is by counts and the determination of differentially methylated cytosines bears strong resemblance to the analysis of RNA-seq data.

In Table 2, we provide comparative summaries of features of COBRA-seq and a number of other methylome sampling methods for DNA methylation analysis.

Specifications such as complexity reduction type and whether they enrich for methylated fractions were compared (Table 2). Genome complexity is reduced by affinity capture (MBDCap-Seq and MeDIP-Seq), restriction digest (COBRA-seq, RRBS, Methyl-Seq, HELP-Seq, CHARM and DREAM) and hybridization capture (Nimblegen SeqCap and Agilent SureSelect). MBDCap-Seq, MeDIP-Seq and COBRA-seq are the only methods that enrich for methylated fragments and hence reduce the sequencing cost, (by yielding a high ratio of CpG information per read sequenced), with the caveat that absolute methylation level estimation is traded for relative methylation level estimation. However, COBRA-seq has an advantage over these other enrichment methods, as the digestion-based enrichment step does not show the same dependence on methylation and CpG density. Therefore, COBRA-seq capture is more uniform across the genome, making it a suitable choice for interrogating regions or genomes of low methylation density. Another system for affinity-based capture of methylated DNA that we have described previously is SuBLiME [42]. Here capture is done after copying of bisulfite-converted DNA to incorporate biotin-dG opposite unconverted meC bases (or biotin-dC in a subsequent copying round). SuBLiME can efficiently capture methylated cytosines in different sequence contexts and can be tuned to different levels of genome coverage [42]. It should be noted that copy number variation in a sample has a multiplicative effect on input DNA. This is observed as a local change in the number of sequenced reads or probe signal intensity on a microarray. With methylation enrichment-based methods, methylation estimates are confounded with copy number and it is only possible to mathematically deconvolute methylation from copy number across large genomic regions.

**Table 2:** Qualitative comparison of selected methylome methods (*are the preferred genomic input, low input will increase variation due to sampling).

Methylome Methods	Complexity Reduction Type	M or M + U Fraction	Methylome Sampling (Yes/No)	Favour of Enrichment Towards	Comments
WGBS [1]	N/A	M + U	N/A	N/A	High cost, can detect non-CpG methylation, genomic input* (0.05–0.1 μg).
MBDCap-Seq [34,62], MIRA-Seq [38]	Affinity capture	M	No	CpG-rich	Dependent on CpG density, effected by salt concentration, covers about 18% of the CpGs [24], 28,500 CpG islands [34], DNA input = 0.2–1 μg.
MeDIP [63], MeDIP-Seq [36]	Affinity capture	M	No	CpG-rich	Bias towards 5mC-rich regions, Captures single-stranded DNA, prone to technical variability, coverage is read-depth dependent, input = 0.15–5 μg.
SuBLiME [42]	Methylated cytosine capture	M	Yes	CpG-rich and poor	Substitutes biotin-14-dCTP or biotin-14-dGTP at the position of the 5mC in bisulfite treated DNA, input = 2 μg.
COBRA-seq	Restriction digest	M	Yes	CpG-rich and poor	Provides relative methylation levels like MeDIP/MBD-Cap, 1%–17% of the CpGs for single digest, can detect non-CpG methylation, input = 0.1–1 μg.
Nimblegen SeqCap [39]	Hybridization capture	M + U	Yes	CpG-rich and poor	“Off-the-shelf” version for human genome only/similar regions covered as 450K array, can be customized [39], can detect non-CpG methylation, input = 0.5–1 μg.
Agilent SureSelect [64]	Hybridization capture	M + U	Yes	CpG-rich and poor	Available for human genome only, covers 3.7 million CpG sites, input = 0.5 μg.
450K array [30,65]	Microarray	M + U	Yes	CpG-rich	Arrays comes in 12 per slide, available only for humans, not readily customized, input = 0.5–1 μg.
RRBS [35,53]	Restriction digest	M + U	Yes	CpG-rich/medium	Can detect non-CpG methylation, input = 0.1–0.3 μg.
Methyl-Seq [33], HELP-Seq [66]	Restriction digest	M + U	Yes	CpG-rich	Assesses 0.25 to 1.3 million CCGG sites in human genome by difference in read fractions in *HpaII vs. MspI* libraries, input = 0.01–0.1 μg.
CHARM [32,67]	Restriction digest	M + U	Yes	CpG-rich and poor	Array-based and available for human, mice and rat, assesses 3.5 to 7 million CpG sites, input = 5 μg.
DREAM [37]	Restriction digest	M + U	Yes	CpG-rich	Assesses methylation at ~0.15 million sites in human genome by sequential *SmaI/XmaI* digestion and library sequencing, input = 5 μg.

In samples with a heterogenous cell population, for example, solid tumour tissue with a high degree of non-neoplastic cells, COBRA-seq presents the same concerns as other methods. For example, with an absolute methylation method, a 70% pure tumour will appear as having a maximum beta of 0.7 (given sufficient read coverage), while for COBRA-seq or other enrichment methods, this will translate to a reduced rate (lambda). Unmethylated regions will still have no reads, but methylated regions will have a reduced rate.

To sequence formalin-fixed paraffin embedded (FFPE) samples, we recommend that researchers optimize the initial sonication step to have fragments between 100–500 bp. FFPE-induced DNA adducts are not a concern for enzyme cutting due to the preceding PCR pre-amplification step. This is further explained in the step-by-step protocol submitted as Supplementary Methods.

The relative coverage of different sequence features by COBRA-seq and selected methylome sampling methods is shown in Figure 5 and Supplementary Table S3. It can be seen that COBRA-Seq using enzymes containing one CpG site provides relative coverage that is very similar to the overall distribution of features in the human genome—CpG islands, CpG shores, promoters, gene bodies and intergenic regions as well as enhancer regions. Use of enzymes containing two CpG sites such as BstUI focuses COBRA-Seq toward more CpG rich regions, islands, shores and promoters. RRBS and DREAM are both strongly biased toward coverage of CpG-rich regions and promoters, but notably, RRBS also covers a high proportion of enhancers. Recently a large *in silico* survey was used to characterise the properties of the RRBS method with other methylation insensitive enzymes [68]. Also, a dual-enzyme RRBS method (dRRBS) using *ApeKI* (5'-G/CWGC) as well as *MspI* has been described [69]. The addition of *ApeKI* offers more representative coverage in low-CG regions without overly fragmenting CpG-rich DNA. The increased coverage by dRRBS is characterized from an *in silico* and empirical standpoint. Because of their current designs, methods relying on hybridization selection of targets, such as 450K arrays and Agilent and Roche/Nimblegen capture systems, are strongly biased toward promoter and CpG-rich regions. However, such capture systems offer the opportunity for designs to evolve and to be specifically targeted to regions of high interest.

## 4. Conclusions

Building upon the previously described COBRA method, the COBRA-seq method described here makes it possible to measure DNA methylation at a large number of specific sites distributed across the genome. Methylome sampling methods broadly utilize two approaches to select the genomic fraction to be analysed. Some methods sample independently of methylation status and measure both unmethylated and methylated cytosines at each CpG site (e.g., 450K array, SeqCap, RRBS and others mentioned in Table 2). Alternatively, other methods specifically enrich the methylated fraction and determine the relative number of reads or level of methylation at different sites across the genome (e.g., MeDIP-Seq, MBDCap-Seq, COBRA-seq). This latter approach is advantageous to reduce cost with less wasted sequencing space and is suited to identification of differences between different samples. Its main drawback is that methylation levels determined are relative, rather than absolute, and can be influenced by the relative efficiency of capture and amplification of different target regions. Because capture of methylated DNA in COBRA-seq is based on restriction digestion and ligation at individual sites, it does not show the same dependence on methylation density as MeDIP-Seq and MBDCap-Seq. making it a suitable choice for interrogating regions or genomes of low methylation density.

In this study, we demonstrated the feasibility of COBRA-seq for generation and analysis of genome-scale DNA methylation profiles at nucleotide resolution and compared the relative methylation measures of COBRA-seq with other methylome sampling methods. COBRA-seq data shows high concordance with WGBS, RRBS and 450K array data, although, these methods vary in the proportions of the genome sampled. *TaqI*-based COBRA-seq does not disproportionally enrich specific genomic features such as promoters and CpG islands and compared to 450K probe density and RRBS *MspI* restriction sites, COBRA-seq *TaqI* sites are uniformly distributed across the genome. Moreover, COBRA-seq is highly adaptable as the principle is compatible with various enzymes, which provides choice for the efficient mapping of 5mCs in any genomic context, including low methylation genomes such as those of insects. The count data produced by COBRA-seq is not well suited for examining methylation within a single sample, instead it is best suited for examining the differences between groups of samples and with modelling of the variance and distribution, *p*-values may be generated in much the same manner as RNAseq count data.

In conclusion, COBRA-seq method has proven to be highly sensitive and samples uniformly across the genome giving better coverage for biomarker discovery studies. COBRA-seq is a unique alternative method to study methylation in low CpG regions such as in enhancers and CpG poor promoters providing unique advantage to the users to finetune the project costs according to budget.

## Supplementary Files

Supplementary File 1
