# Supplementary material for: COBRA-Seq: Sensitive and Quantitative Methylome Profiling
Source: Genes (Basel). 2015 Oct 23;6(4):1140–63. doi: 10.3390/genes6041140 (PMC4690032; doi:10.3390/genes6041140)
Supplement: Supplementary File 1 [file genes-06-01140-s001.pdf]

Supplementary Materials

1. Supplementary Figures

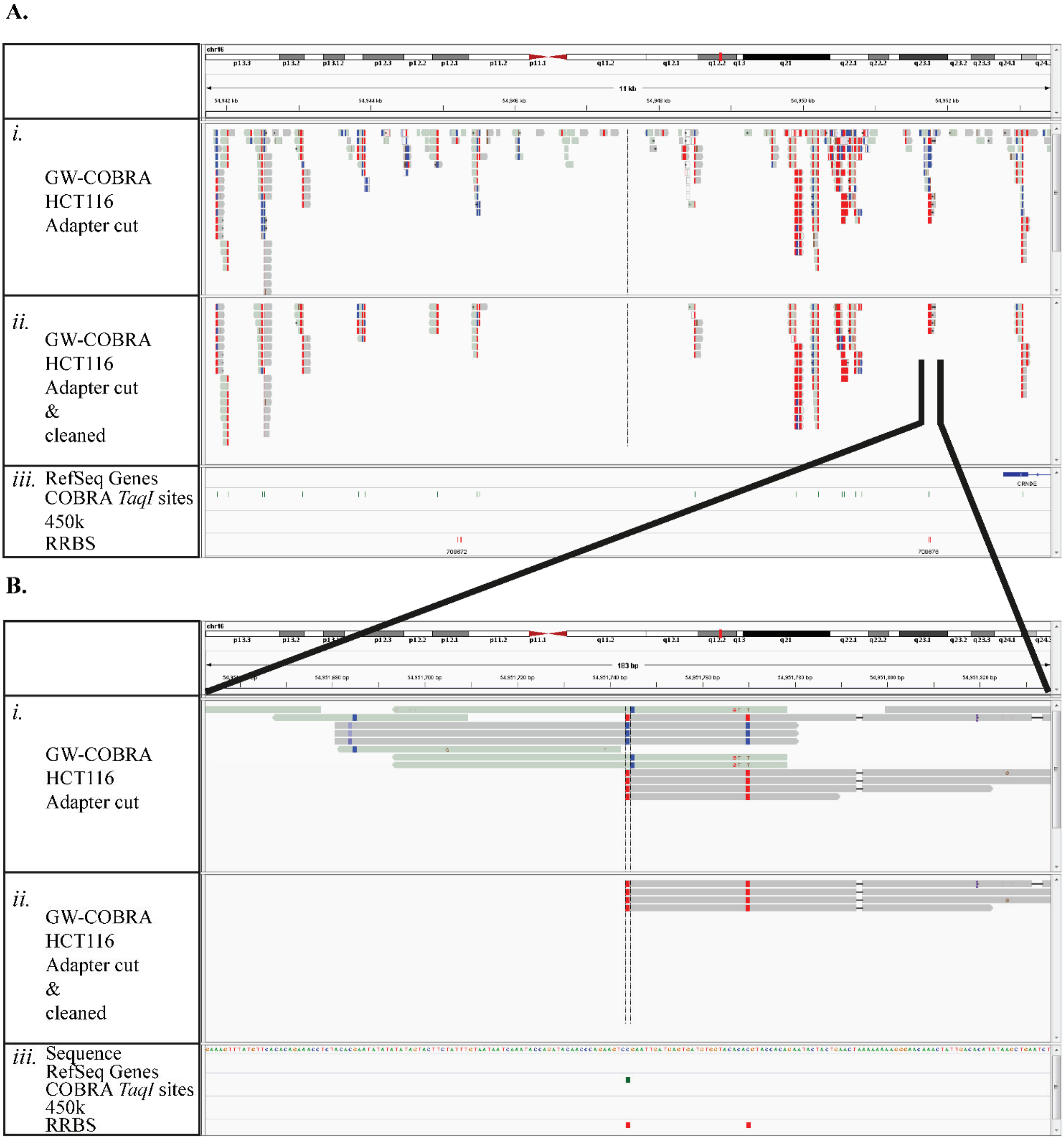

Figure S1. *Cont.*

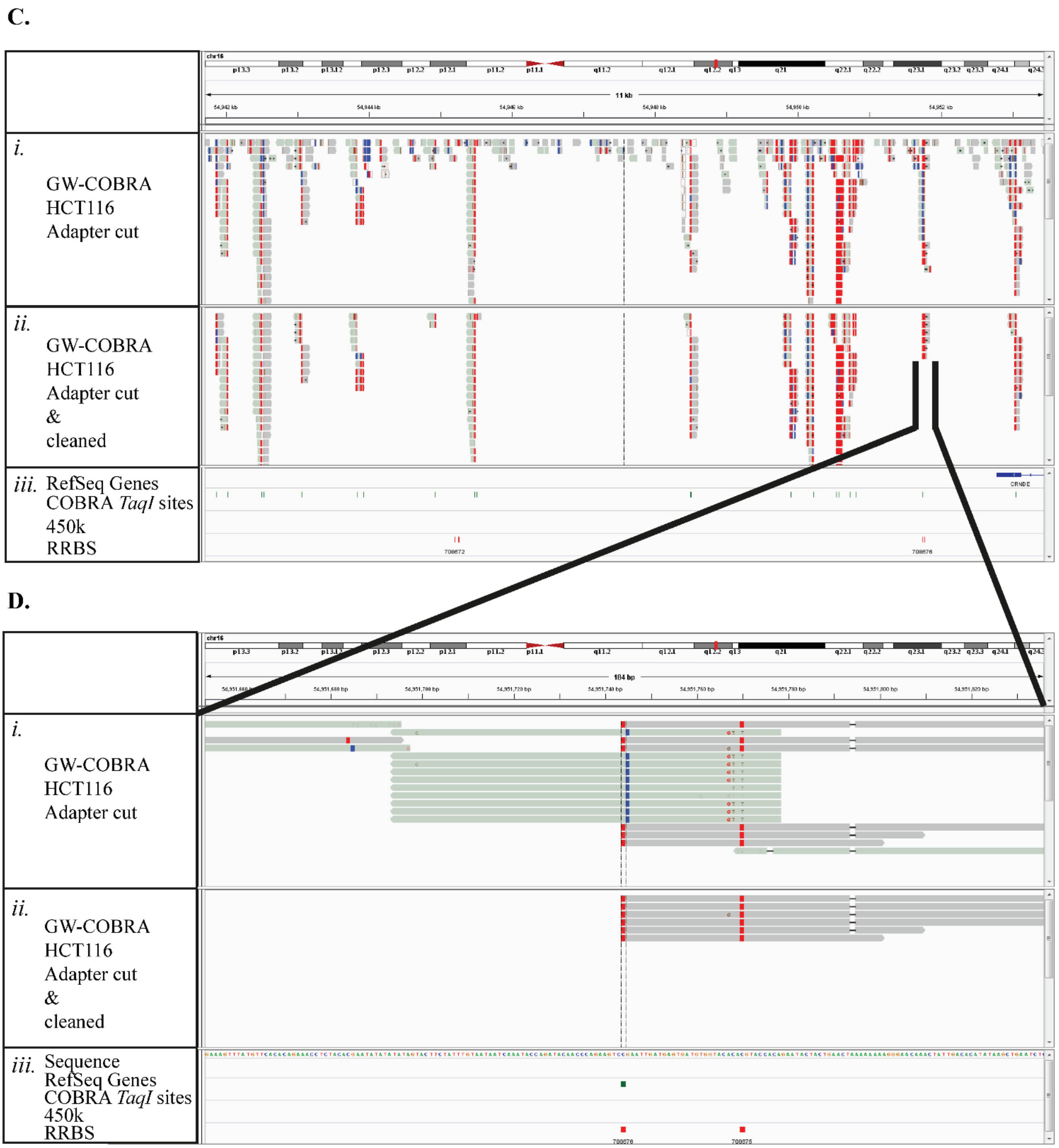

Figure S1. IGV screenshots of (**A**) GW-COBRA cleaning process presented in 11 kb window *CRNDE* gene; (**B**) GW-COBRA cleaning process presented in 183 bp window near *CRNDE* gene; (**C**) LA-COBRA cleaning process presented in 11 kb window *CRNDE* gene; (**D**) LA-COBRA cleaning process presented in 183 bp window *CRNDE* gene.

Fragment Preparation:
100 bp< fragments <500 bp, end polished, phosphorylated, Poly A-tailed DNA sample

```
5'-pNNNN.........NNNNA-3'
3'-ANNNN.........NNNNp-5'
```

Ligation of Adapter 2:
5-methylcytosines are shown as 5 whereas unmehtylated Cytosines are shown as C

```
5'-TGT5A55G5TGGT5AT5CGCT
                        \
                         GCTCTTCCGATCTNNNN.........NNNNAGAT5GGAAGAG5T5GTATG55GT5TT5TG5TTG-3'
 3'-GTT5GT5TT5TG55GTATG5T5GAGAAGG5TAGANNNN.........NNNNTCTAGCCTTCTCG
                                                                    \
                                                                     TCGC5TA5TGGT5G55A5TGT-5'
```

Bisulfite Conversion:
Fragments are single stranded (cytosines were converted to uracils while5-methylcytosines were maintained as cytosines)

```
5'-TGTCACCGCTGGTCATCUGUTGUTUTTUUGATUTNNNN.........NNNNAGATCGGAAGAGCTCGTATGCCGTCTTCTGCTTG-3'
```

GW-COBRA Limited PCR Amplification:
Cycle 1 (A single primer will be used in the first round of PCR as the upper and lower strands are same)

```
5'-TGTCACCGCTGGTCATCUGUTGUTUTTUUGATUTNNNN.........NNNNAGATCGGAAGAGCTCGTATGCCGTCTTCTGCTTG-3'
   ..............................................3'-TCTAGCCTTCTCGAGCATACGGCAGAAGACGAAC-5'
```

Initial Product (both same)

```
5'-TGTCACCGCTGGTCATCUGUTGUTUTTUUGATUTNNNN.........NNNNAGATCGGAAGAGCTCGTATGCCGTCTTCTGCTTG-3'
3'-ACAGTGGCGACCAGTAGACAACAAAAAAACTAAANNNN.........NNNNTCTAGCCTTCTCGAGCATACGGCAGAAGACGAAC-5'
```

Cycles 2-6

```
5'-TGTCACCGCTGGTCATCUGUTGUTUTTUUGATUTNNNN.........NNNNAGATCGGAAGAGCTCGTATGCCGTCTTCTGCTTG-3'
   ..............................................3'-TCTAGCCTTCTCGAGCATACGGCAGAAGACGAAC-5'

5'-bTGTCACCGCTGGTCATCTGTTGTTT-3'...............................................
 3'-ACAGTGGCGACCAGTAGACAACAAAAAAACTAAANNNN.........NNNNTCTAGCCTTCTCGAGCATACGGCAGAAGACGAAC-5'
```

Final Product of GW-COBRA Limited PCR amplification

```
5'-bTGTCACCGCTGGTCATCTGTTGTTTTTTGATTTNNNN.........NNNNAGATCGGAAGAGCTCGTATGCCGTCTTCTGCTTG-3'
 3'-ACAGTGGCGACCAGTAGACAACAAAAAAACTAAANNNN.........NNNNTCTAGCCTTCTCGAGCATACGGCAGAAGACGAAC-5'
```

Figure S2. *Cont.*

LA-COBRA Limited PCR Amplification:
Cycle 1 (A single primer will be used in the first round of PCR as the upper and lower strands are same)

```
5'-TGTCACCGCTGGTCATCUGUTGUTUTTUUGATUTNNNN.........NNNNAGATCGGAAGAGCTCGTATGCCGTCTTCTGCTTG-3'
   ........................................................... 3'-CGAGCATACGGCAGAAGACGAACAGGGATATCACTCAGCATAATTTAAG-5'
```

Initial Product (both same)

```
5'-TGTCACCGCTGGTCATCUGUTGUTUTTUUGATUTNNNN.........NNNNAGATCGGAAGAGCTCGTATGCCGTCTTCTGCTTG-3'
3'-ACAGTGGCGACCAGTAGACAACAAAAAAACTAAANNNN.........NNNNTCTAGCCTTCTCGAGCATACGGCAGAAGACGAACAGGGATATCACTCAGCATAATTTAAG-5'
```

Cycles 2-6

```
5'-TGTCACCGCTGGTCATCUGUTGUTUTTUUGATUTNNNN.........NNNNAGATCGGAAGAGCTCGTATGCCGTCTTCTGCTTG-3'
    ..............................................................3'-CGAGCATACGGCAGAAGACGAACAGGGATATCACTCAGCATAATTTAAG-5'

5'-bTGTCACCGCTGGTCATCTGTTGTTT-3'.....................................................................................
3'-ACAGTGGCGACCAGTAGACAACAAAAAAACTAAANNNN.........NNNNTCTAGCCTTCTCGAGCATACGGCAGAAGACGAACAGGGATATCACTCAGCATAATTTAAG-5'
```

Final Product of LA-COBRA Limited PCR amplification

```
5'-bTGTCACCGCTGGTCATCTGTTGTTTTTTGATTTNNNN.........NNNNAGATCGGAAGAGCTCGTATGCCGTCTTCTGCTTGTCCCTATAGTGAGTCGTATTAAATTC-3'
3'-ACAGTGGCGACCAGTAGACAACAAAAAAACTAAANNNN........NNNNTCTAGCCTTCTCGAGCATACGGCAGAAGACGAACAGGGATATCACTCAGCATAATTTAAG-5'
```

Restriction Digest: GW-COBRA material *TaqI*

Figure S2. Extended COBRA-seq scheme for constructing both GW-COBRA and LA-COBRA sequencing libraries.

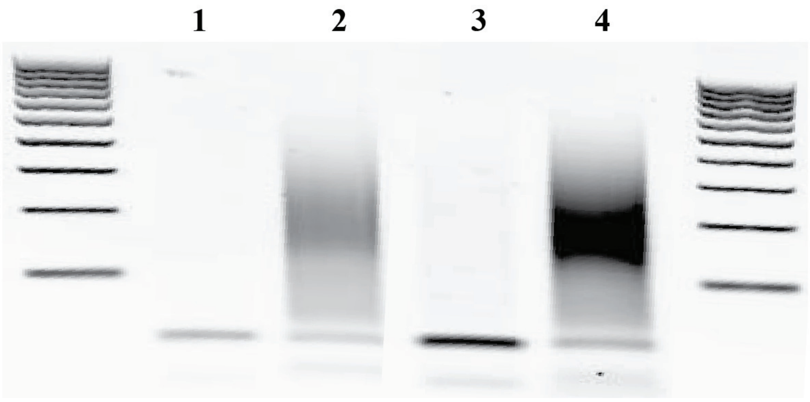

Figure S3. COBRA-seq library construction with minimal starting genomic DNA material. 3% low melting agarose gel and stained with SYBR gold. Adapter-2 ligated and bisulfite-treated genomic DNA amplified as described in limited PCR amplification step. 0.1 and 1 µg of starting genomic DNA material amplified using GW-A2 Fwd and Rev primers respectively (lane 2 and 4). Lane 1 and 3 are negative controls for the limited PCR amplification step.

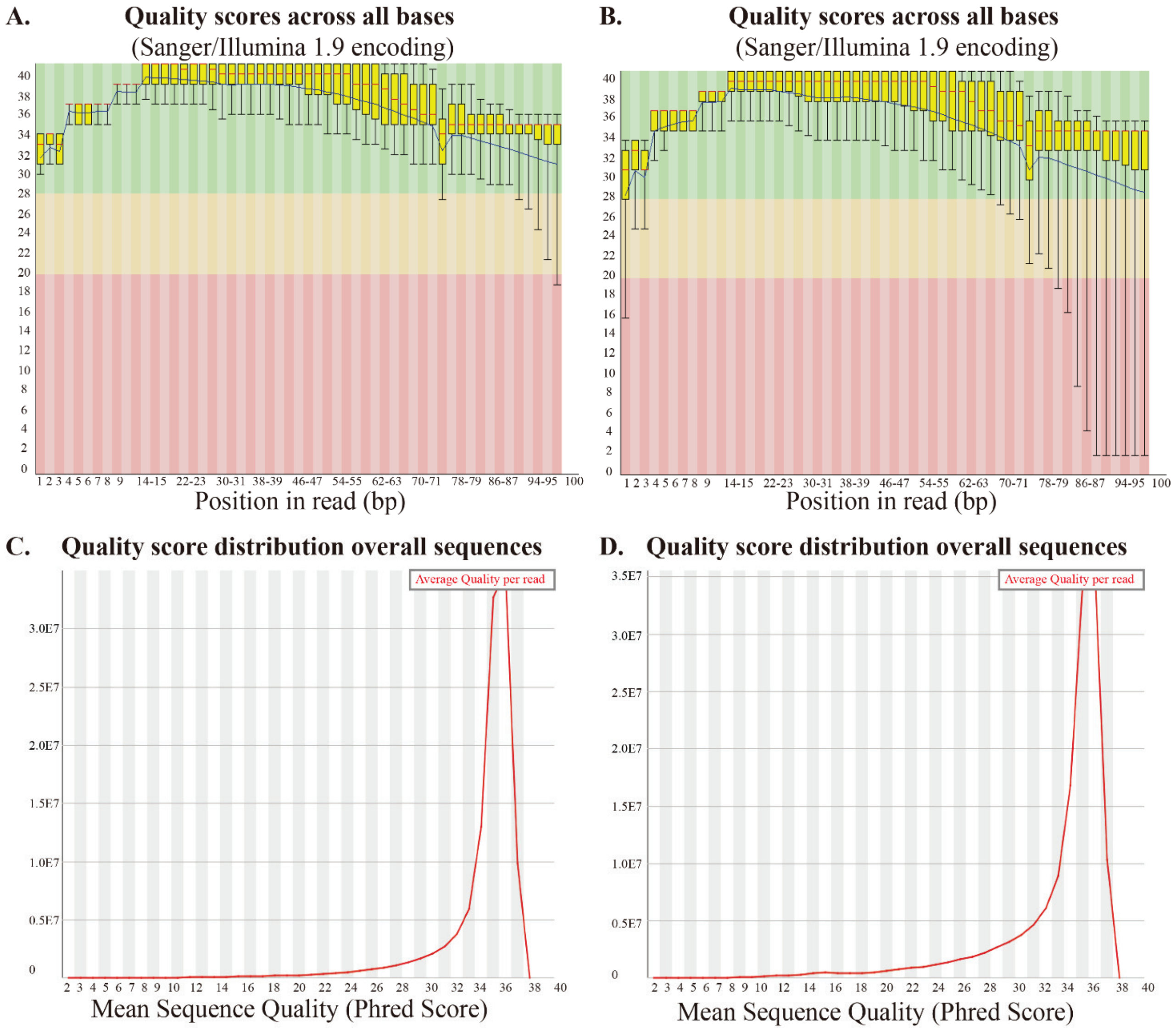

Figure S4. *Cont.*

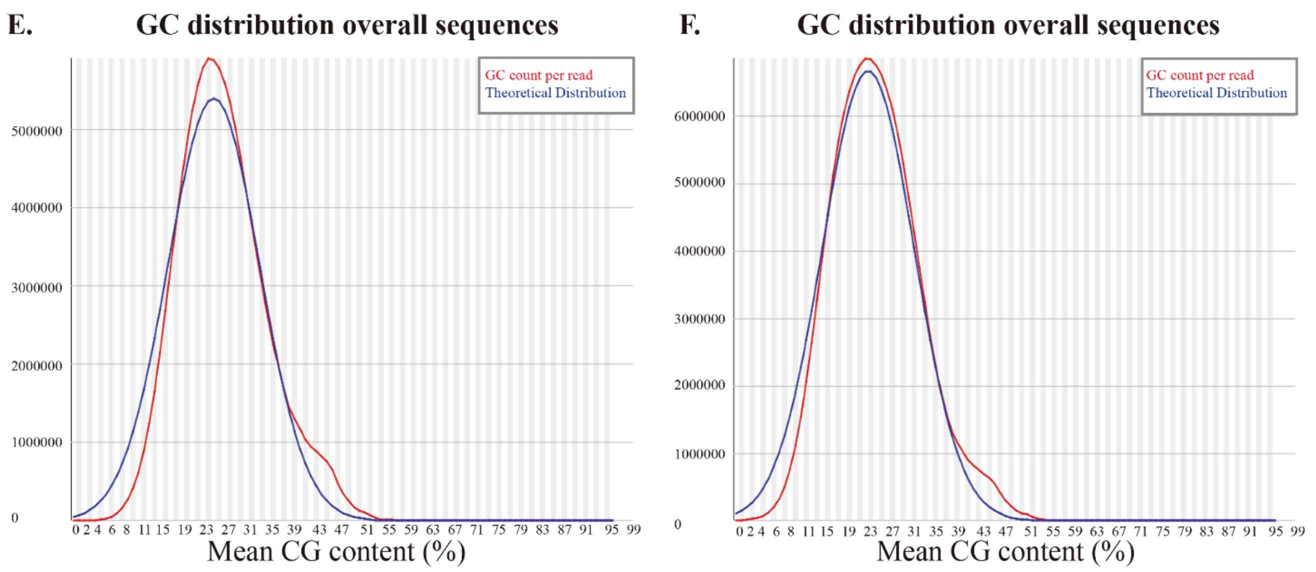

Figure S4. FastQC summary reports of sequencing reads. (**A**) and (**B**) Quality scores across all bases for GWCOBRA and LA-COBRA sequencing results respectively; **(C)** and (**D**) Quality score distribution of overall GW-COBRA and LA-COBRA sequences respectively; (**E**) and (**F**) GC distribution of overall GW-COBRA and LA-COBRA sequences respectively.

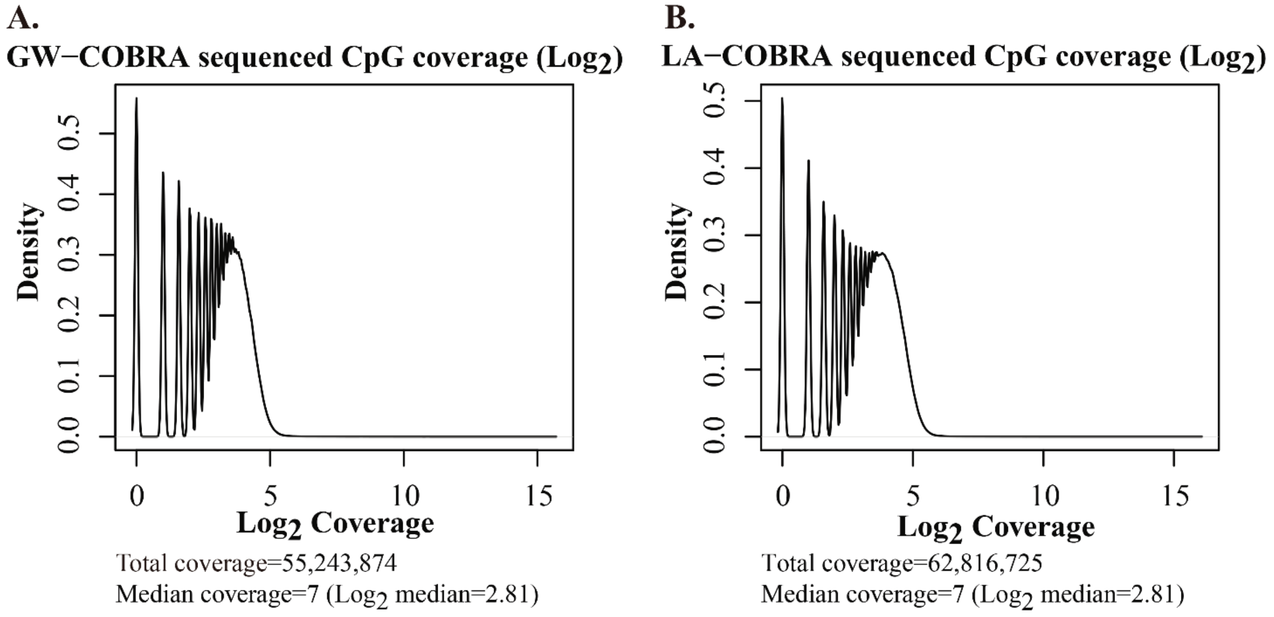

Figure S5. *Cont.*

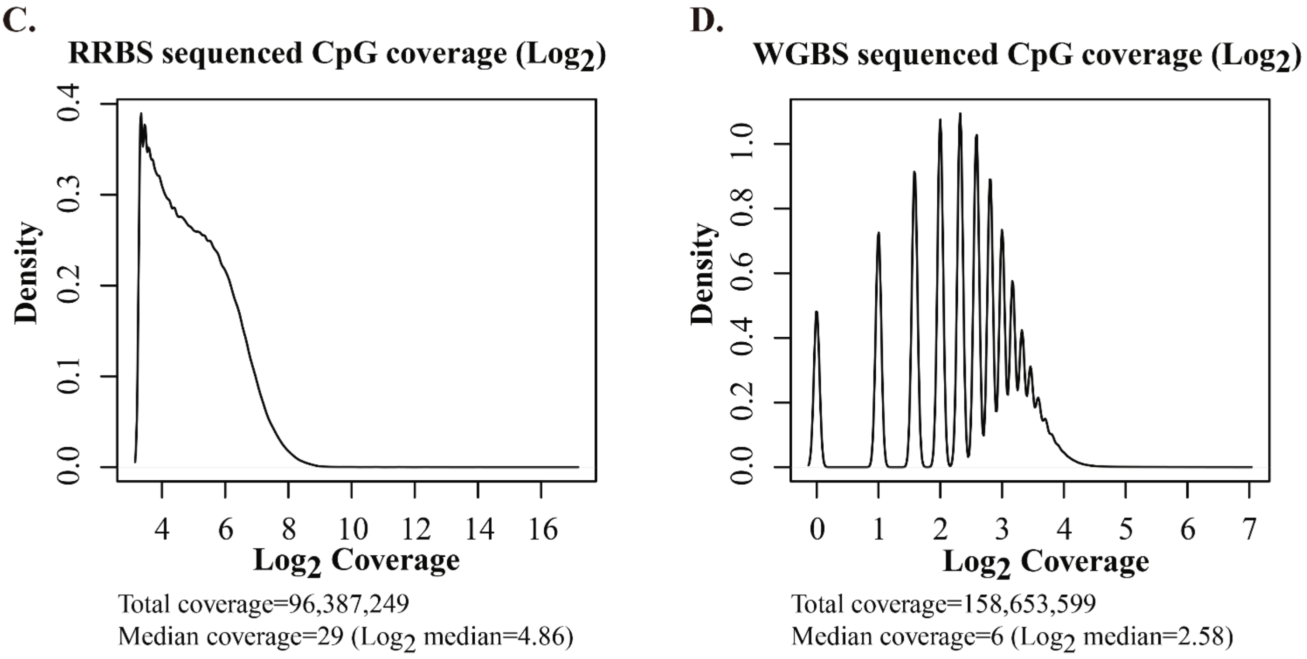

Figure S5. Density plots of the CpG site coverage in the HCT-116 GW- and LA-COBRA libraries and public RRBS and WGBS libraries. Coverage has been transformed by Log2 as each method has a small fraction of CpG sites with factors of magnitude more coverage than the median coverage. (**A**) GW-COBRA; (**B**) LA-COBRA; (**C**) RRBS and (**D**) WGBS.

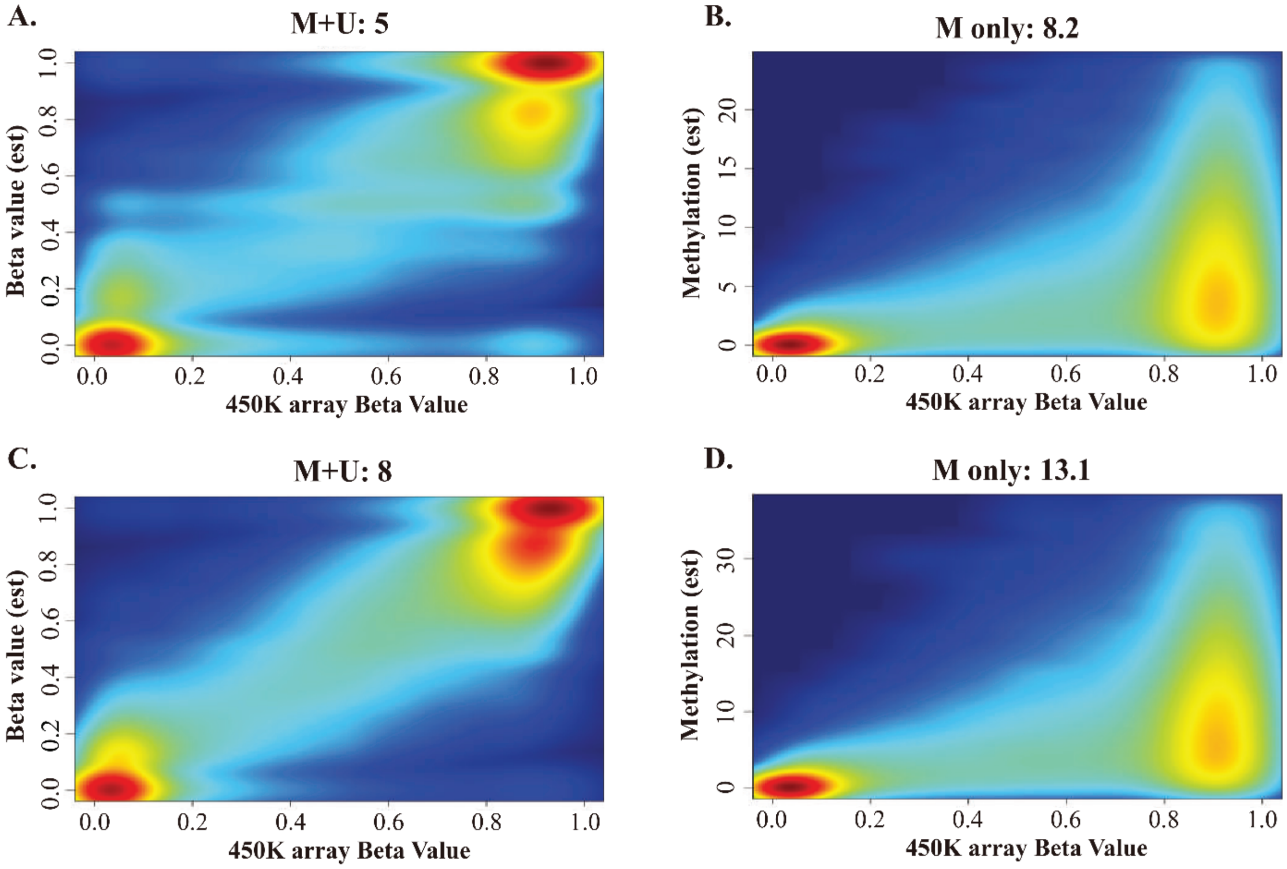

Figure S6. *Cont.*

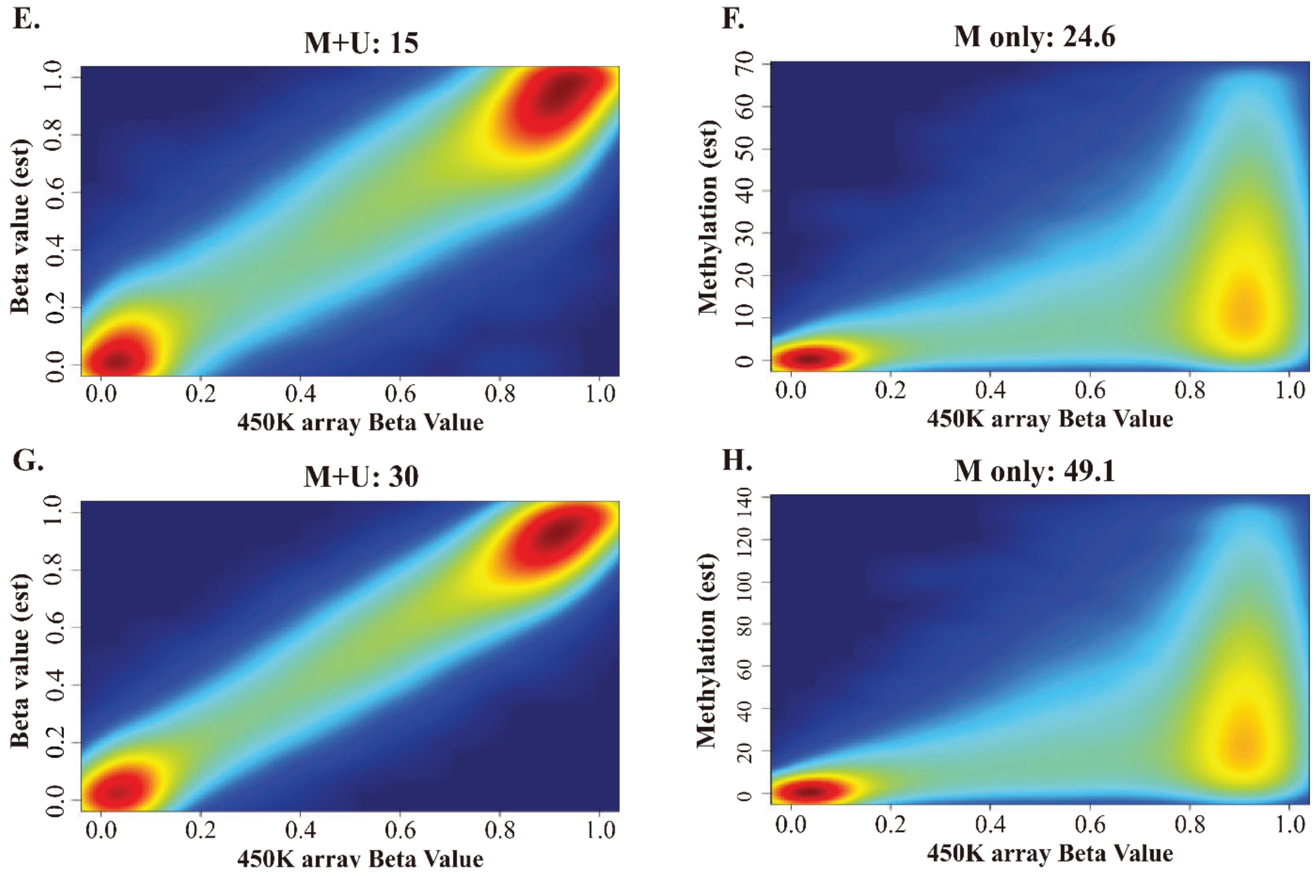

Figure S6. Simulation of methylome sampling methods that targets methylated (M) only or both M and unmethylated (U) fractions. (**A**) Population mean (λ) coverage of 5 in M+U fractions; (**B**) Population mean (μ) coverage of 8.2 in M fraction only (**C**) λ coverage of 8; (**D**) μ coverage of 13.1; (**E**) λ coverage of 15; (**F**) μ coverage of 24.6; (**G**) λ coverage of 30 and (**H**) μ coverage of 49.1.

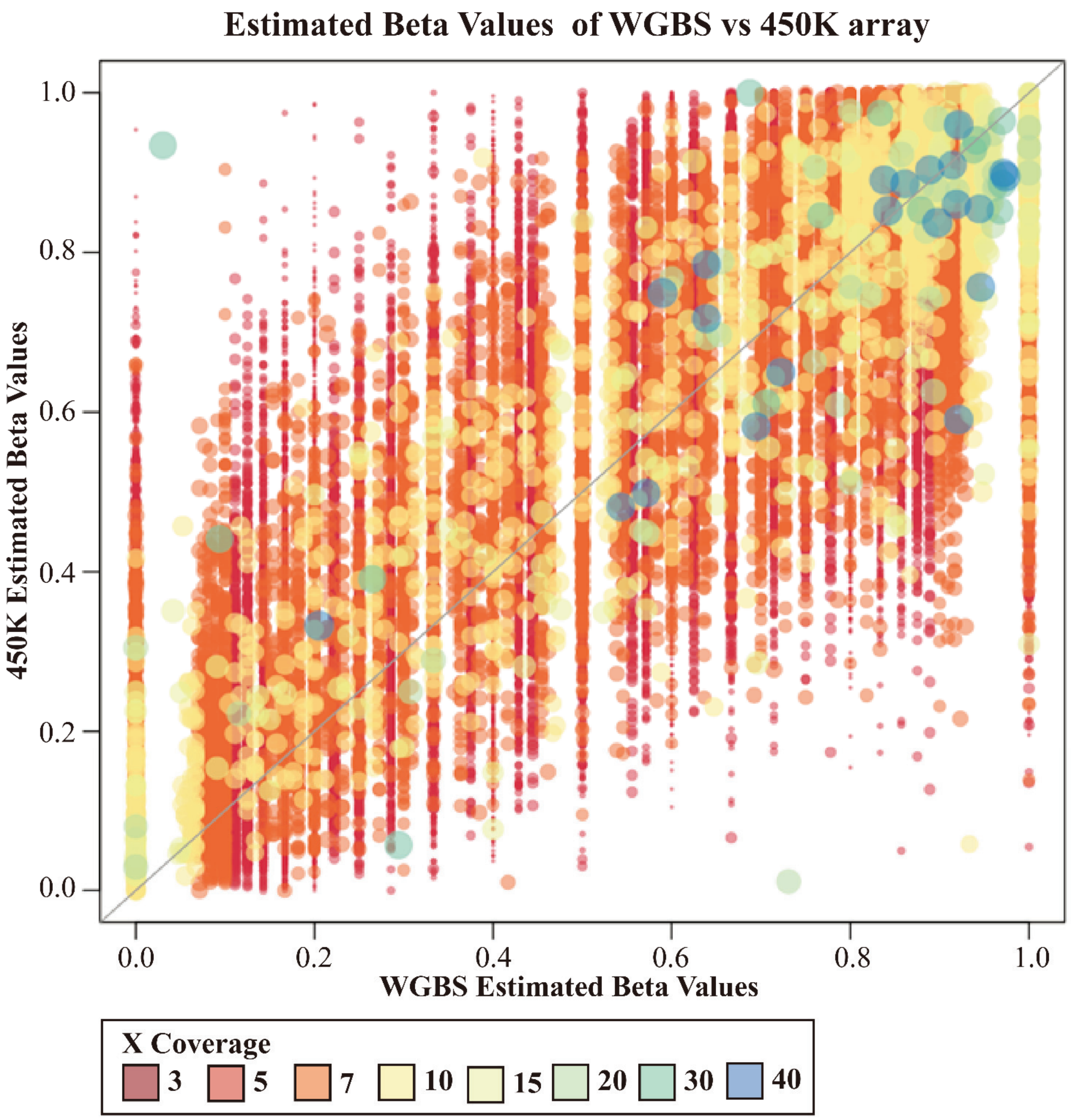

Figure S7. An empirical comparison mapping 200,000 HCT-116 450K array beta values to WGBS beta values.

A.

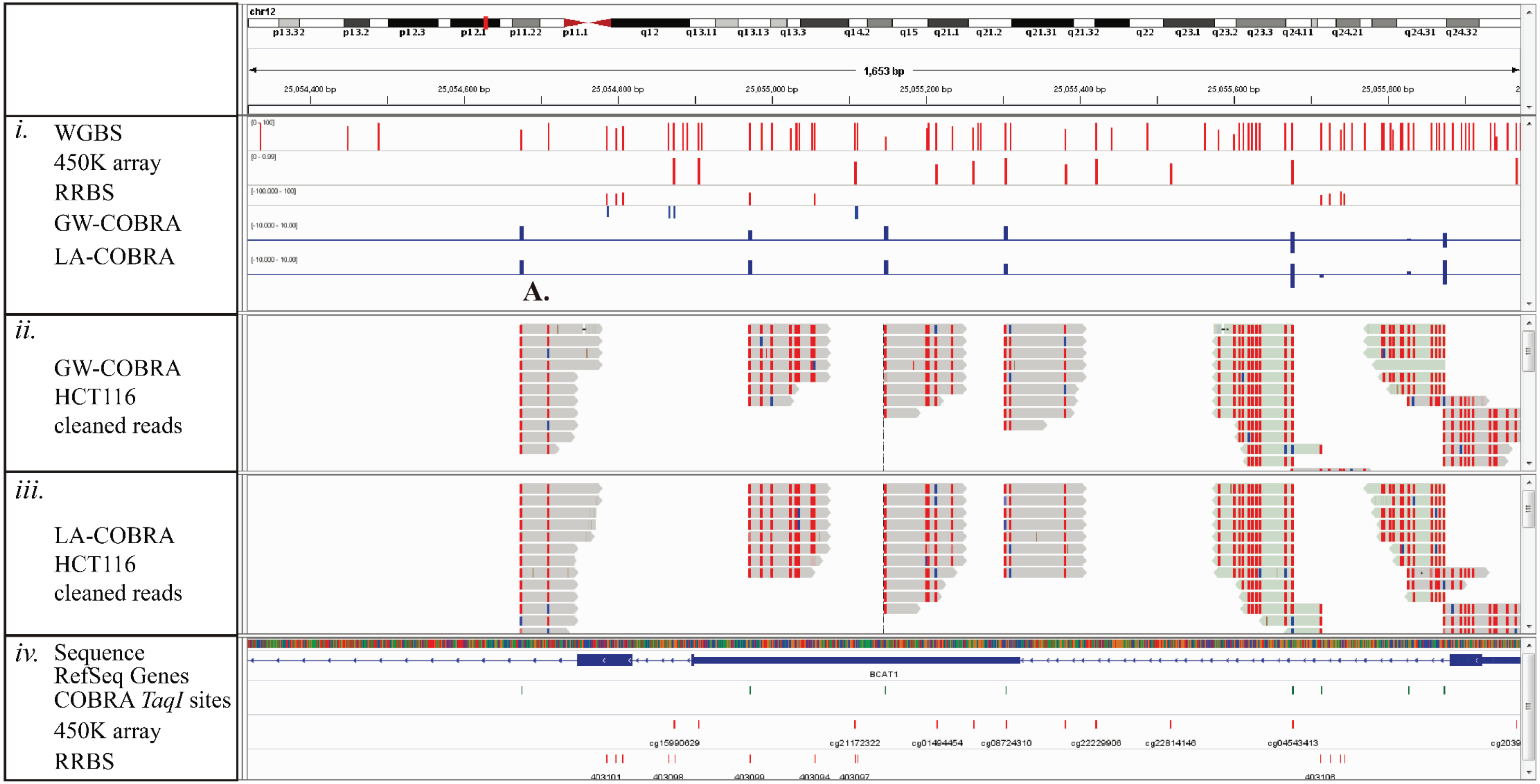

Figure S8. *Cont.*

B.

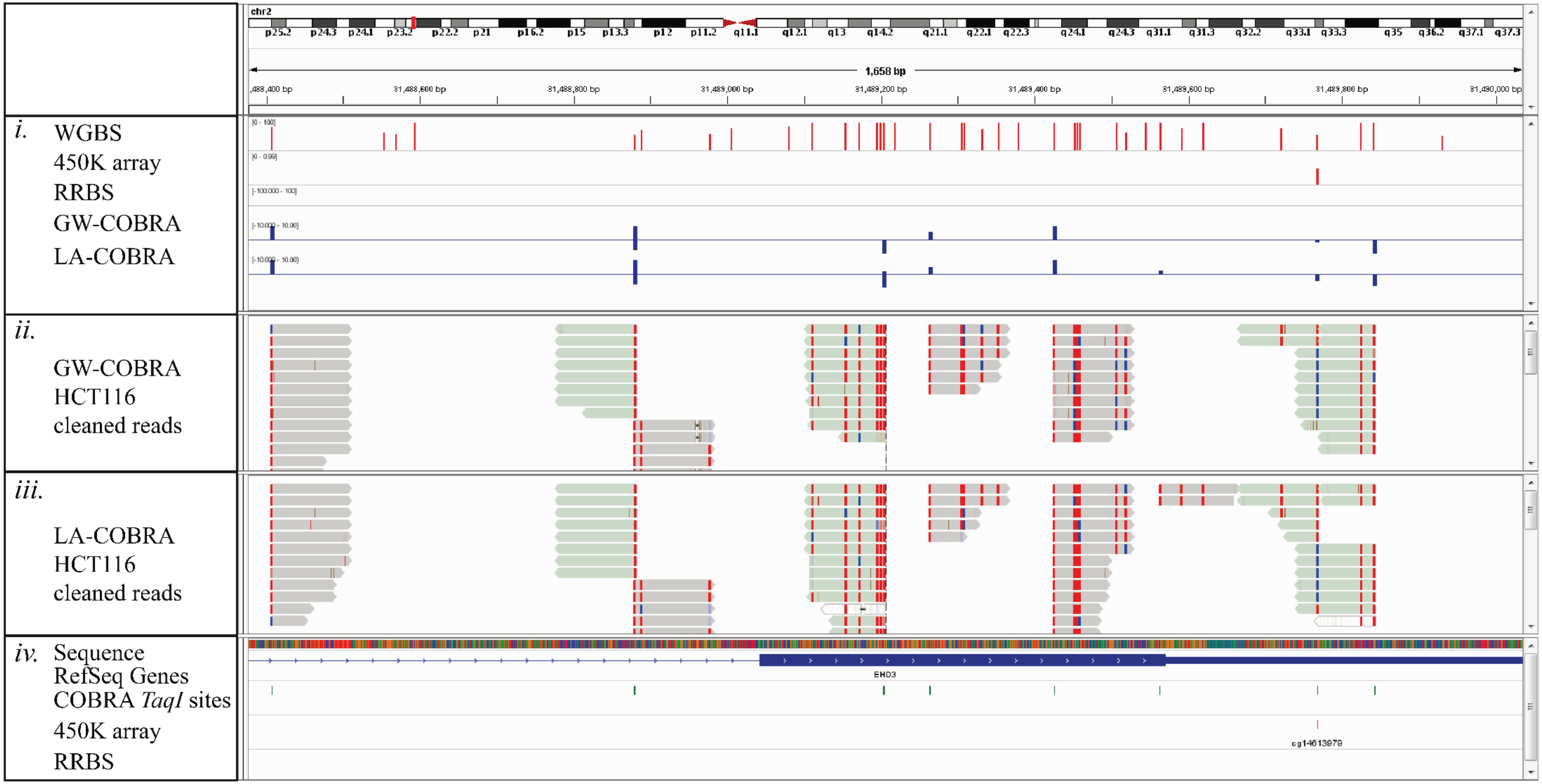

Figure S8. IGV screenshots are to the selected colon cancer associated genes to display read stacks: (**A**) *BCAT1*; (**B**) *EHD3*. *i*. Methylation levels determined by WGBS, 450K arrays, RRBS, GW-COBRA and LA-COBRA respectively; *ii*. Genome coverage and stacking of cleaned GW-COBRA reads; *iii*. Genome coverage and stacking of cleaned LA-COBRA reads; *iv*. Genomic locations of accessible COBRA *TaqI* sites, 450K array probes and accessible RRBS sites.

A.

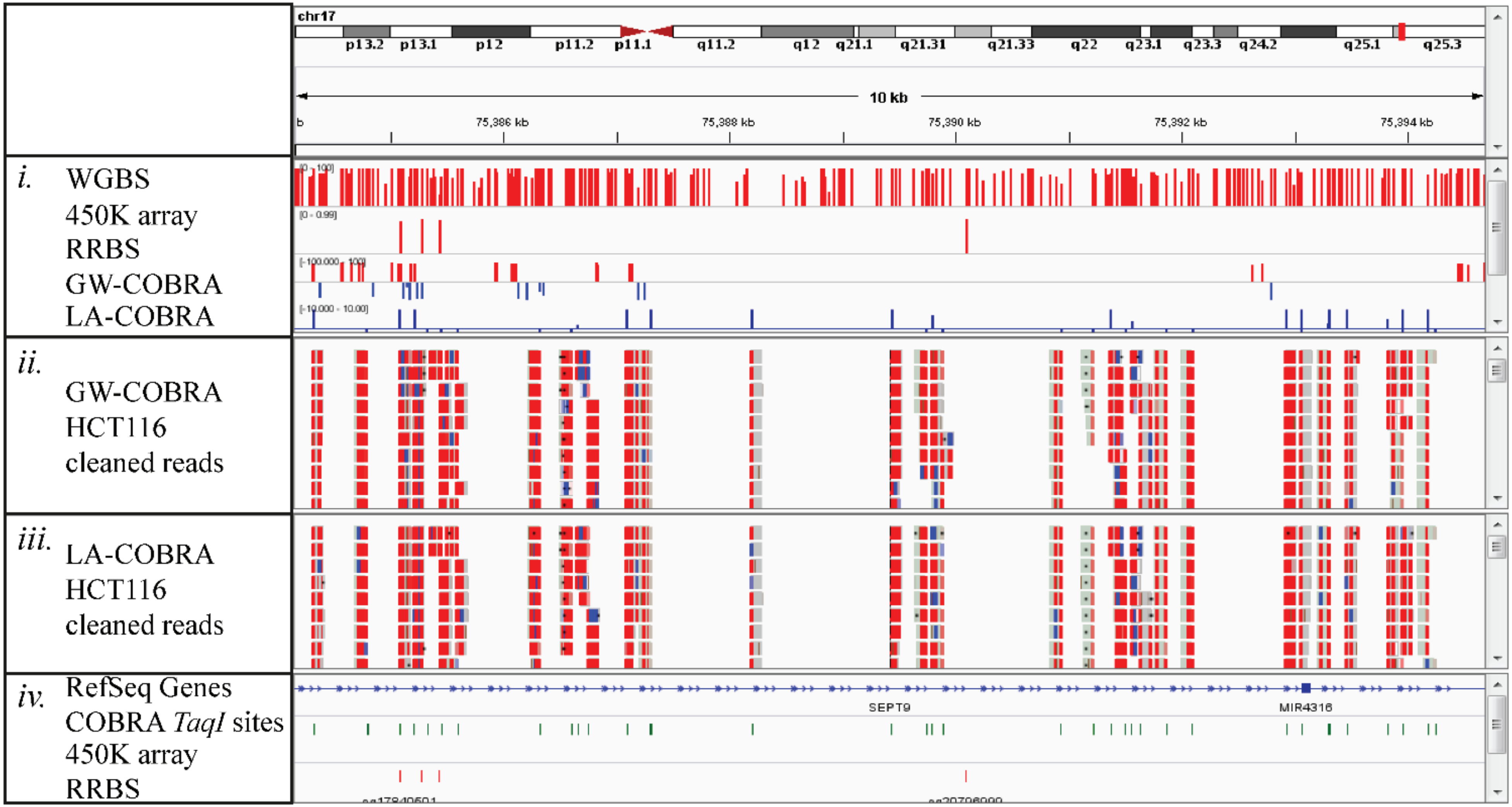

Figure S9. *Cont.*

B.

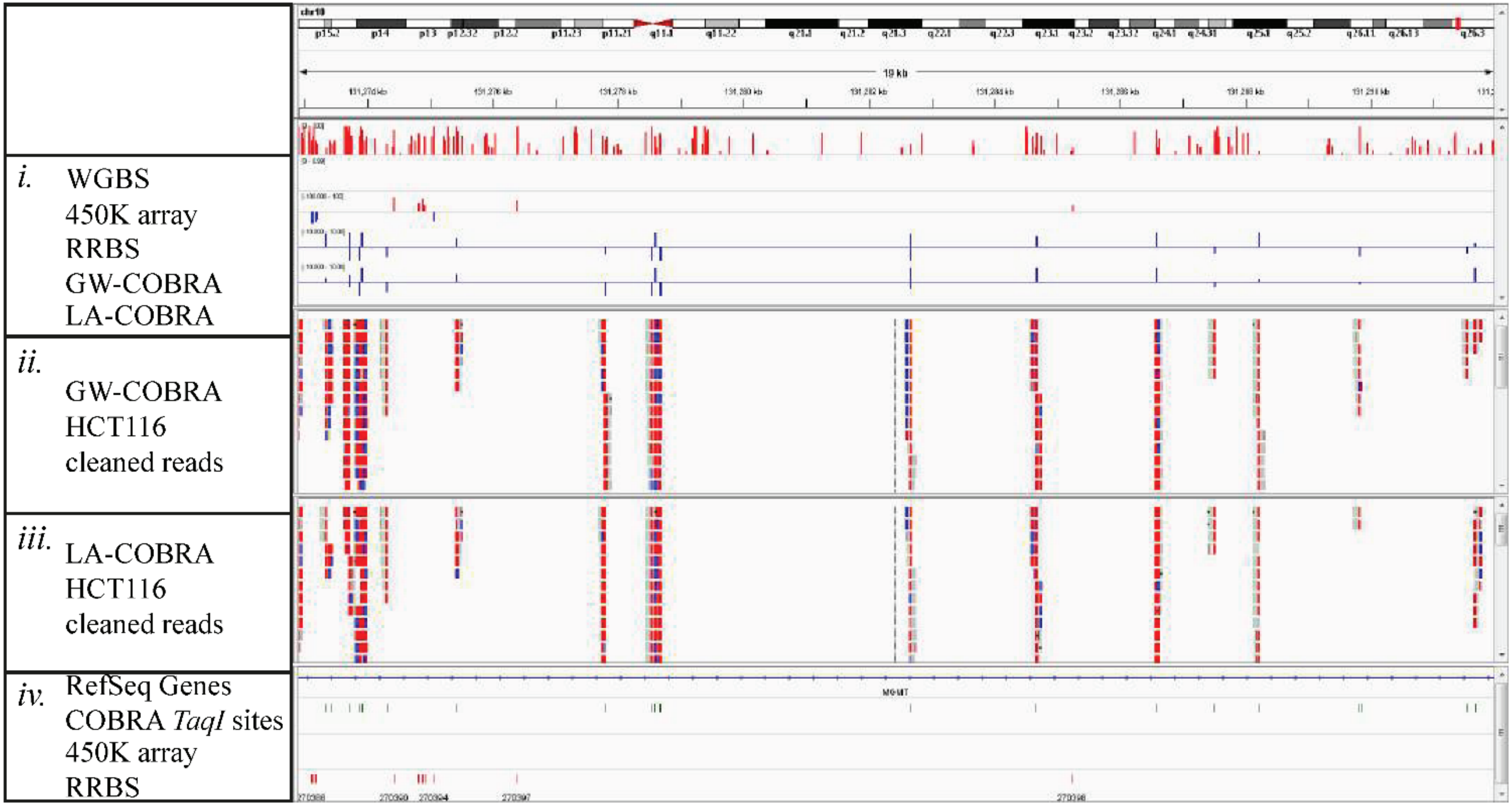

Figure S9. *Cont.*

C.

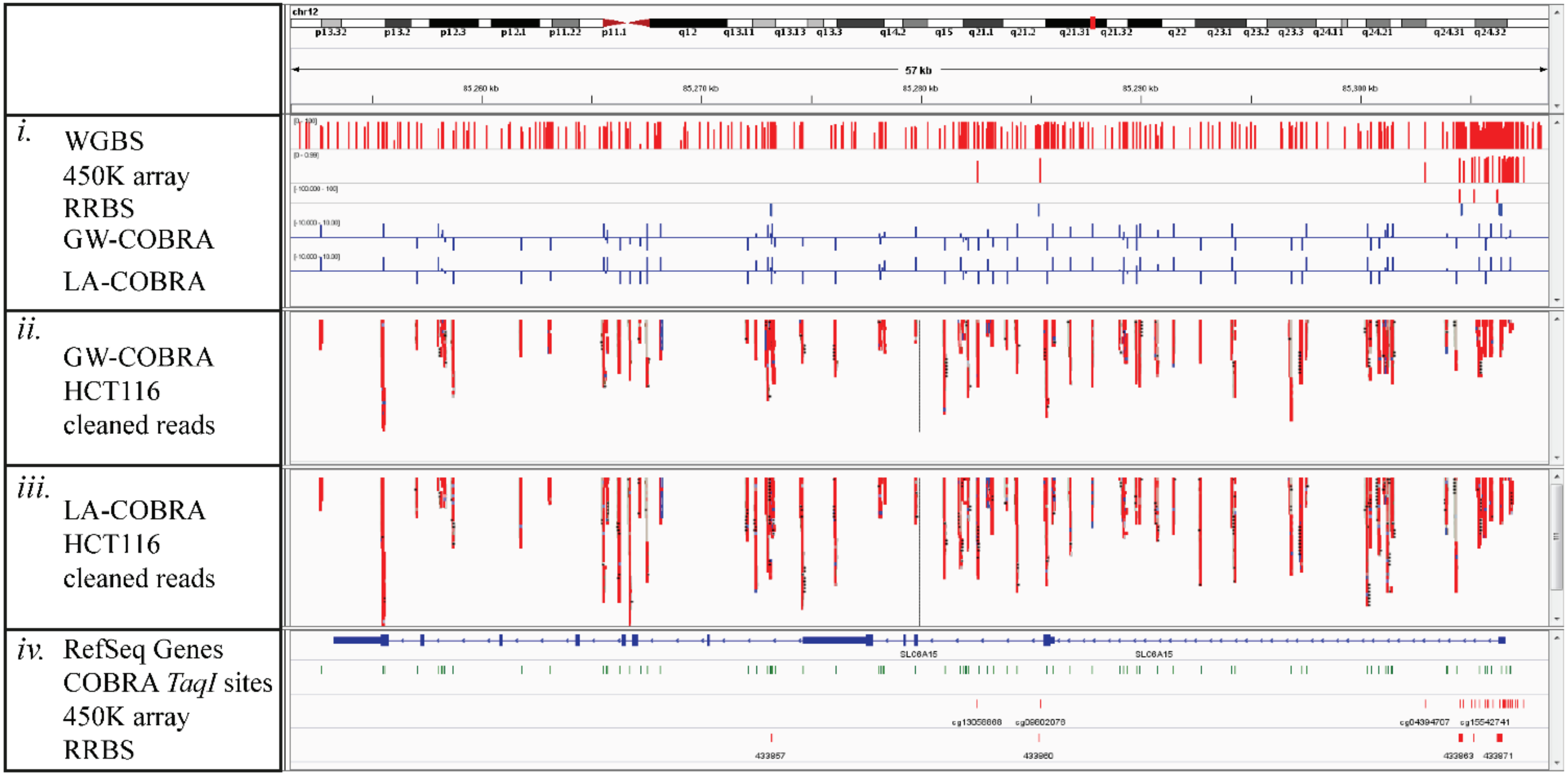

Figure S9. *Cont.*

D.

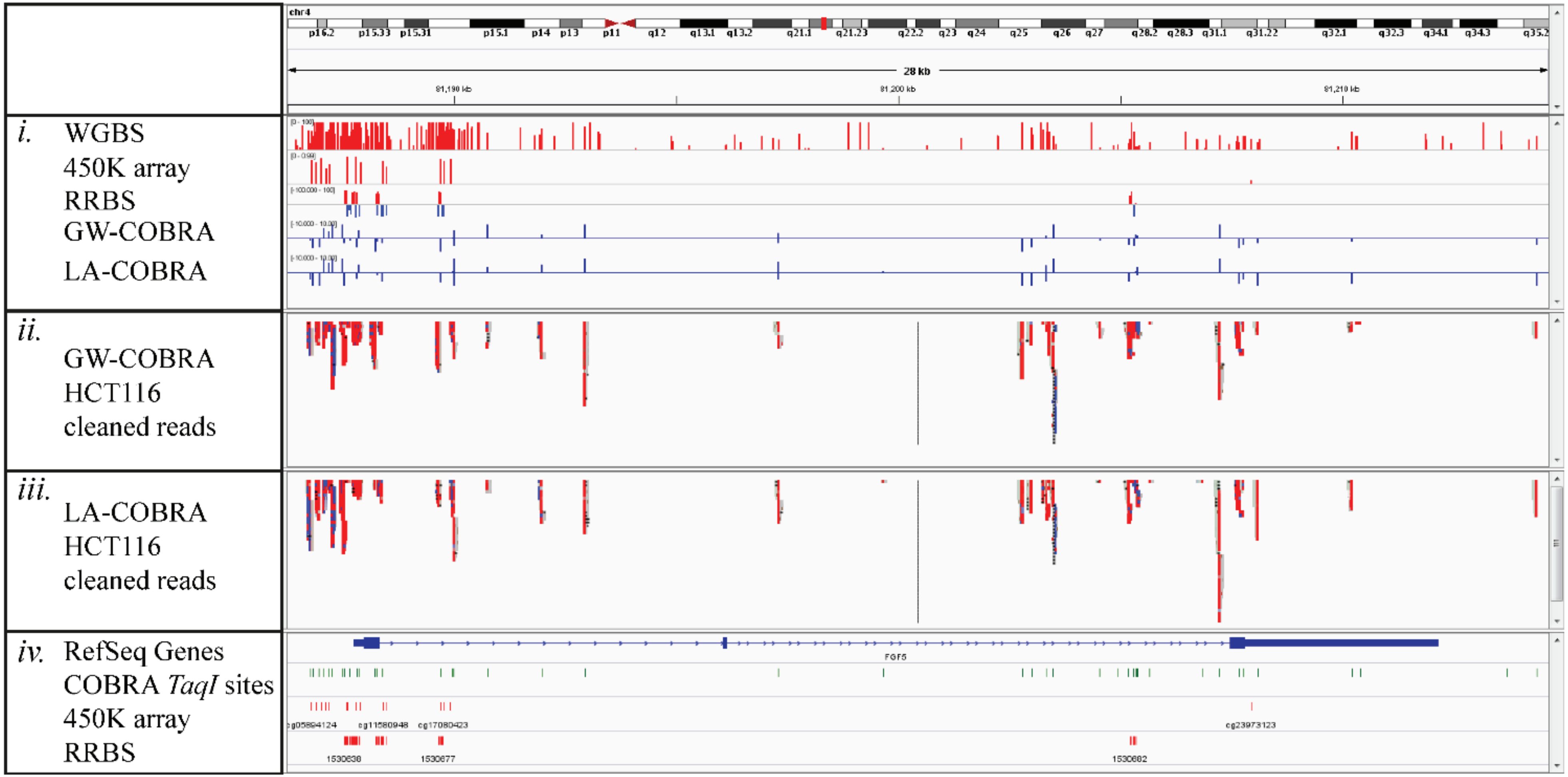

Figure S9. IGV screenshots of methylation profiles on selected colon cancer associated genes: (**A**) *SEPT9*; (**B**) *MGMT*; (**C**) *SLC6A15* and (**D**) *FGF5*. *i*. Methylation levels determined by WGBS, 450K arrays, RRBS, GW-COBRA and LA-COBRA respectively; *ii*. Genome coverage and stacking of cleaned GW-COBRA reads; *iii*. Genome coverage and stacking of cleaned LA-COBRA reads; *iv*. Genomic locations of accessible COBRA *TaqI* sites, 450K array probes and accessible RRBS sites.

2. Supplementary Tables

Table S1. GW-COBRA and LA-COBRA sequencing read statistics.

**Stage**	**Statistic**	**GW-COBRA**	**LA-COBRA**
Raw file	Read Counts	115,097.029	142,245,797
BAM file	Total BAM file entries	117,215,649	145,687,410
	Unique Read IDs	97,410,988	122,495,417
	%Unique entries	83.1	84.1
Mapping	Original Mapped	108,463,362	134,521,871
	Original Unmapped	8,752,287	11,165,539
	Forward	62,852,260	78,337,754
	Reverse	54,363,389	67,349,656
	% Mapped	92.53%	92.34%
Filtering	Converted to Unmapped Total	55,043,953	73,715,118
	Reason		
	Secondary alignment	11,052,374	12,026,454
	Start Sequence not *TaqI* site	27,499,711	44,507,174
	Not within *in silico* reference	53,219,488	71,705,146
	Not in reference, but could rescue		
	Not in ref, has *TaqI* site	31,633,261	33,795,306
	Not in ref, has *TaqI* site and primary	28,761,986	30,945,333

Table S2. Complexity reduction statistics.

Methylation Context	Enzyme	Recognition Site (5'-3')	Overhang	Restriction Sites	Total Fragments	Sequenceable Fragments	Total CpG Coverage
CpG	*HpyCH4IV*	A/CGT	CG-5'	5,192,248	7,343,344	6,506,928	4,776,485
	TaqI	T/CGA	CG-5'	4,977,110	6,489,977	5,756,193	4,486,417
	AclI	AA/CGTT	CG-5'	692,617	855,037	840,839	683,730
	ClaI	AT/CGAT	CG-5'	388,259	473,628	467,368	384,128
	BstBI	TT/CGAA	CG-5'	866,821	972,174	953,489	850,949
	PvuI	CGAT/CG	3'-AT	46,118	58,497	57,392	90,550
	BstuI	CG/CG	Blunt	725,727	1,451,454	1,058,938	1,209,854
	MluI	A/CGCGT	CGCG-5'	75,894	97,019	93,919	147,648
	BsiWI	C/GTACG	GTAC-5'	90,219	101,772	96,998	172,337
	Hpy99I	CGWCG/	3'-CGWCG	555,835	710,425	556,444	863,001
CpG and CpN	*HinP1I*	G/CGC	CG-5'	490,520	537,572	426,631	802,697
CpN	*CviQI*	G/TAC	TA-5'	1,493,098	1,504,651	1,444,102	1,476,629
	BfuCI/Sau3AI	/GATC	GATC-5'	1,295,990	1,308,369	1,269,237	1,294,261
	EcoRI	G/AATTC	AATT-5'	368,461	369,242	366,551	375,916
CnG	*HpyCH4III*	ACN/GT	3'-N	1,497,255	1,497,255	1,444,743	1,444,743
	Hpy188I	TCN/GA	3'-N	1,371,760	1,371,760	1,298,590	1,212,237
Other Methylome Methods	SuBLiME (*CviQI*)			5,047,091	10,094,182	8,702,060	21,388,229
	DREAM (*XmaI*)			374,921	749,842	51,046	497,412
	RRBS (*MspI*)			2,297,221	4,594,442	1,528,440	4,724,246
	450K array			-	-	-	484,022

Table S3. Complexity reduction statistics cont'd. The % of genome coverage per genomic location by COBRA-seq and other genome sampling methods.

	Transcript	Exon	TSS (4 kb)	Genebody	Intergenic	CpG Island	CpG Shore	CpG Ocean	5' UTR	3' UTR	DNaseI HS	Enhancer	Repeat	Total CpGs
Genome-wide	52.3	7.5	12.8	43.0	44.2	7.4	7.2	85.4	1.8	1.4	26.5	0.7	52.3	28,217,448
HpyCH4IV	50.4	4.8	8.2	44.6	47.3	2.5	5.7	91.8	0.8	1.3	21.8	0.7	52.0	4,776,485
TaqI	50.9	6.2	9.6	43.9	46.5	3.5	6.3	90.2	1.1	1.3	21.5	0.6	55.2	4,486,417
AclI	49.2	5.1	8.2	43.3	48.5	2.2	5.8	92.0	0.8	1.5	23.2	0.7	41.1	683,730
ClaI	48.4	6.5	7.8	42.6	49.7	2.3	5.1	92.6	0.9	1.4	20.9	0.5	43.2	384,128
BstBI	50.9	5.6	9.1	44.3	46.6	2.7	6.2	91.1	0.9	1.2	18.6	0.5	56.6	850,949
PvuI	57.9	17.7	28.6	36.4	35.0	24.8	9.8	65.4	5.7	1.8	44.4	1.1	36.2	90,550
BstuI	56.9	13.1	27.1	37.3	35.6	24.0	10.4	65.7	4.7	1.6	38.9	1.1	52.4	1,209,854
MluI	57.4	13.0	24.5	39.8	35.7	21.4	10.1	68.5	3.9	1.9	40.4	1.2	43.7	147,648
BsiWI	58.2	14.1	23.5	41.1	35.4	20.5	9.6	70.0	3.9	1.8	40.5	1.1	42.5	172,337
Hpy99I	59.6	20.7	31.9	35.6	32.5	30.2	10.6	59.3	6.6	2.1	49.9	1.3	33.0	863,001
HinP1I	56.8	12.3	28.6	36.5	34.8	25.6	9.6	64.8	5.1	1.3	35.9	1.0	61.4	802,697
CviQI	52.7	6.6	10.3	45.1	44.6	4.8	6.4	88.7	1.1	1.5	25.0	0.7	51.9	1,476,629
BfuCI/Sau3AI	52.1	6.7	11.0	44.0	45.0	5.0	6.6	88.4	1.4	1.3	21.9	0.6	59.3	1,294,261
EcoRI	51.0	4.3	9.2	44.5	46.3	3.0	6.4	90.6	0.8	1.0	14.6	0.4	72.9	375,916
HpyCH4III	51.9	6.7	9.3	44.9	45.7	3.5	6.5	90.1	1.0	1.5	25.7	0.7	47.2	1,444,743
Hpy188I	52.3	9.7	14.0	41.9	44.1	7.4	8.2	84.4	2.1	1.7	32.6	0.9	40.8	1,212,237
SuBLiME	52.5	7.4	11.7	43.8	44.4	6.1	6.6	87.2	1.6	1.5	24.8	0.7	53.0	21,388,229
DREAM	63.5	24.8	53.5	24.1	22.4	54.2	13.6	32.2	11.3	1.7	64.4	1.5	32.8	497,412
RRBS	59.4	17.8	33.4	34.7	31.9	30.4	12.7	56.9	6.4	1.9	46.7	1.2	44.9	4,724,246
450K array	61.6	23.1	51.2	29.8	19.0	31.1	23.2	45.6	8.4	4.6	66.8	1.7	15.8	484,022

3. Supplementary Materials and Methods

3.1. COBRA-Seq Library Preparation Protocol in Detailed

Part 1: Construction of Adapters

Each Adapter was made using two oligonucleotides dissolved in sterile water with a final concentration of 500 µM (Table 1). The Adapter-2 oligonucleotides partially complement each other therefore the adapter has a "Y" shape. Particularly after bisulfite treatment Adapter-2 upper strand remains unconverted and non-complementary to Adapter-2 lower strand On the other hand all the Adapter-1 combinations were designed using fully complementary oligonucleotides.

P1.1. Mix the matching oligonucleotides with 1× NEB Quick Ligation Buffer in a final concentration of 50 µM each and in a final volume of 100 µL.

P1.2. Hybridize the oligonucleotides with the following temperature cycles on a PCR machine: 95 °C 5 min, 72 °C 5 min, 60 °C 5 min, 50 °C 3 min, 40 °C 3 min, 30 °C 3 min, 20 °C 3 min and 10 °C 3 min. Upon hybridisation the adapters were stored at −20 °C.

P1.3. Store them in a cool rack at −20 °C.

Part 2: Sequencing Library Preparation Protocol

Part 2-1. DNA Fragmentation

P2-1.1. Resuspend genomic DNA in 300 µL low TE to a final concentration of 16.66 ng/µL.

P2-1.2. Fragment the genomic DNA using a Bioruptor UCD-200 sonicator (Diagenode, Belgium) at a power setting of "high" for four sets of 30 cycles of 15 s on/off with 15 min intervals on ice in between each set.

P2-1.3. For visualisation, run 5 µL of the sonicated material on 3% Biorad Low Range Ultra Agarose gel.

P2-1.4. The fragments range should be between 100–500 bp.

Part 2-2. DNA Precipitation

P2-2.1. To precipitate the fragmented DNA, add 600 µL volumes of cold, absolute ethanol, 30 µg of GlycoBlue and 100 µL of 3 M sodium acetate.

P2-2.2. Vortex the samples, quickly spin at full speed and incubate at −20 °C for 20 min.

P2-2.3. Centrifuge the samples at full speed (16,000 g) for 15 min at 4 °C.

P2-2.4. Decant the supernatant and wash the blue pellet with 70% cold ethanol.

P2-2.5. Air dry the residual ethanol for 5 min and samples.

P2-2.6. Resuspend the pellet in 36 µL low TE.

P2-2.7. Measure the DNA concentration was measured using 1 µL in NanoDrop 1000 Spectrophotometer.

Part 2-3. End Repair

The End-It Repair reaction kit (Epicentre, Wisconsin) was used to convert DNA with incompatible overhangs such as 5'-protruding and/or 3'-protruding ends to blunt-ended, 5'-phosphorylated DNA by exploiting the 5' to 3' polymerase and 3' to 5'exonuclease enzymes.

P2-3.1. The sonicated and precipitated library material was used with 5 µL 10× End-Repair Buffer, 5 µL dNTP mix, 5 µL ATP and 1 µL Enzyme mix, in a final volume of 50 µL.

P2-3.2. Incubate the reaction at room temperature for 45 min then heat activate the enzymes at 70 °C for 10 min.

Part 2-4. Reaction Clean up

P2-4.1. Increase the volume of the End Repair reaction to 200 uL using sterile water.

P2-4.2. Add an equal volume of phenol: chloroform: isoamyl alcohol.

P2-4.3. Vortex the samples for 15 s and spin at 16,000 g for 5 min.

P2-4.4. Transfer the upper aqueous phase to a new 2 mL microfuge tube.

P2-4.5. Add Sodium acetate to have a final salt concentration of 0.3 M.

P2-4.6. Add three volumes of cold, absolute ethanol, together with 2 µL of GlycoBlue.

P2-4.7. Invert the samples, quickly spin and incubate at −20 °C for 20 min.

P2-4.8. Centrifuge the samples at full speed (16,000 g) for 15 min at 4 °C.

P2-4.9. Decant the supernatant and wash the blue pellet with 70% cold ethanol.

P2-4.10. Air dry the residual ethanol for 5 min and samples.

P2-4.11. Resuspend the pellet in 42 µL low TE.

P2-4.12. Measure the DNA concentration was measured using 1 µL in NanoDrop 1000 Spectrophotometer.

Part 2-5. A-Tailing

A single adenine nucleotide was added to the 3' ends of the blunt fragments to facilitate ligation with adapters that have T overhang, and to limit ligating to one another during the adapter ligation reaction.

P2-5.1. Mix the end repaired library material with 5 µL of 10× NEBNext dA-Tailing reaction buffer (10 mM Tris-HCl, 10 mM $MgCl_2$, 50 mM NaCl, 1 mM DTT, 0.2 mM dATP pH 7.9) and 3 µL of 3' to 5' exonuclease.

P2-5.2. Incubate at 37 °C for 30 min, then heat inactivate the enzyme at 75 °C for 20 min and cool on ice prior to ligation.

Part 2-6. Adapter-2 Ligation

Adapter-2 were designed to contain a corresponding single thymine nucleotide on the 3' end which provides a complementary overhang to ligate to the genomic library fragments (Figure S1).

P2-6.1. Prepare the Ligation reaction with 2 µg of fragmented DNA, 10 molar excess Adapter-2 in a 50 µL reaction containing 2000 U T4 Ligase Enzyme and 25 µL Quick Ligation Buffer.

P2-6.2. Incubate the reaction at room temperature for 20 min and cool the samples on ice.

Note: The ten molar excess can be calculated considering the library fragments average 200 bp and contain two ligatable ends.

There are **X** moles of ligatable ends in the sonicated material,

$$\mathbf{X} = 2\ \mu\text{g DNA} \times 2\ \text{(two ends)}/(650\ \text{Da/bp} \times 200\ \text{bp}) = 3.1 \times 10^{-5} = 31\ \text{pmol}$$

Adapter-2/Fragment = 10:1 31 pmol × 10 = 310 pmol

Therefore, 10 molar excess is 6.2 µL of the 50 µM Adapter-2 stock for 2 µg library material.

Part 2-7. Removal of Excess Adapter-2

P2-7.1. Mix one volume of ligation reaction with 5 volumes of Buffer PB supplied in Qiagen Quick PCR purification kit.

P2-7.2. Apply the mixture into a MinElute column and centrifuge at full speed for 1 min.

P2-7.3. Run the flow-through through the MinElute column again before discarding.

P2-7.4. Wash the column with 750 µL Buffer PE and centrifuged for 1 min.

P2-7.5. Wash the column again with 500 µL Buffer PE and centrifuge for 1 min.

P2-7.6. To remove the residual ethanol, place the column into an empty collection tube and spin when the lid is open at full speed for 1 min.

P2-7.7. Elute the library material twice in 42 µL Elution Buffer with 1 min centrifugation.

Part 2-8. Check point: PCR Reaction

Although this step was not necessary for the GW-COBRA and LA-COBRA library preparation, it was designed as a control point to determine the efficiency of Adapter-2 ligation step which is crucial to have a well distributed library in the range of 200–500 bp. The library material is expected to contain Adapter-2 in both ends, therefore the single primer was employed as a forward and reverse primer during this amplification step.

P2-8.1. Perform the PCRs in 25 µL volume using GoTaq white (Promega) in the buffer supplied with the enzyme, with the addition of 0.2 µM final concentration of a single primer targeting one strand of the Adapter-2.

P2-8.2. The temperature cycles for the PCR are: 95 °C for 3 min; 95 °C for 15 s, 60 °C for 30 s and 72 °C for 1 min, 8 cycles; a final extension of 72 °C for 2 min.

P2-8.3. Run the PCR product to access the success of Adapter-2 ligation on 3% Low Range Ultra Agarose (Biorad).

Note: Figurative representation of Check point PCR step is provided in Figure S10.

It is likely that, short fragments (100–200 bp) may form a hairpin structure or ligate to one another (Figure 2). Over amplification will create library artefact and resulting higher MW bands on an agarose gel. It is recommended to use maximum of 10 PCR cycles.

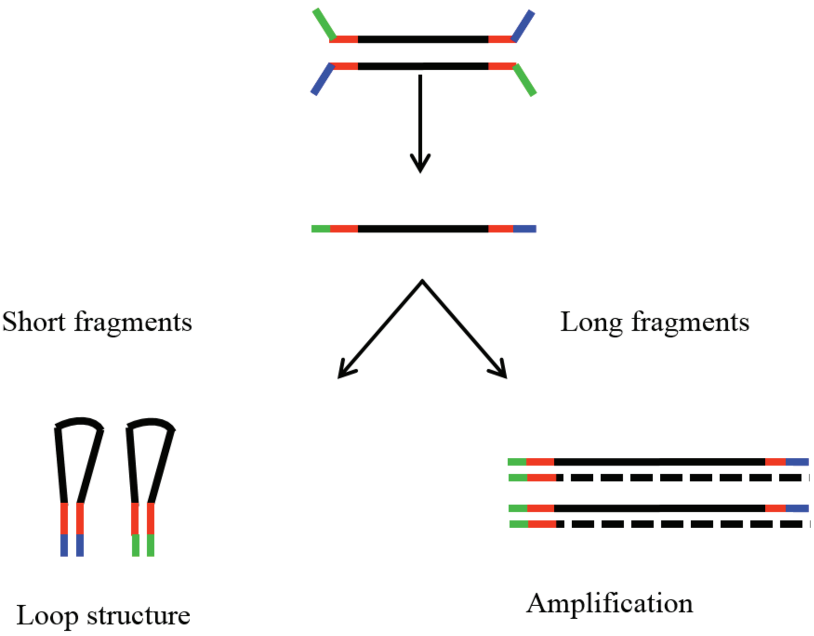

Figure S10. Schematic representation of products generated by the minimal PCR step.

Part 2-9. Bisulfite Treatment

Bisulfite treatment, also known as bisulfite conversion, is a gold standard method for DNA methylation analysis. Bisulfite deaminates unmethylated cytosine to produce uracil whereas methylated cytosines are protected. The locations of unmethylated cytosines and 5-methylcytosines can be determined at single-nucleotide resolution via sequencing.

P2-9.1. Ligated fragments containing Adapter-2 in both ends were bisulfite treated using an EZ DNA Methylation-Gold Kit (Zymo Research).

P2-9.2. Prepare aliquots of the 10 µg/µL library material in a final volume of 20 µL in PCR tubes and add 130 µL of the CT Conversion Reagent for the bisulfite treatment step.

P2-9.3. Incubate the bisulfite conversion reaction at 99 °C 5 min, 60 °C 25 min, 99 °C 5 min, 60 °C 85 min, 99 °C 5 min, 60 °C 175 min and 22 °C 5 min.

P2-9.4. Wash the reaction and desulphonate as per manufacturer's instruction.

P2-9.5. Elute the final product twice in 16 µL.

P2-9.6. Quantify Single stranded DNA concentration using 1 µL in NanoDrop 1000 Spectrophotometer with RNA settings.

Post-bisulfite treatment, the Adapter-2 oligonucleotides are not complementary. At this step, the library material is single stranded.

Part 2-10. Minimal PCR Amplification (Biotin Tagging)

Single stranded bisulfite treated library materials are used as templates with a final concentration of 1 ng/µL in 25 µL using GoTaq white (Promega, Madison, WI, USA) enzyme and buffer mixture. The primers are used in the final concentration of 0.1 µM. The reverse primer contains 5' double biotin whereas the LA-COBRA forward primer had an overhang of T7 promoter region that is used to generate RNA copies via *in vitro* transcription that are subsequently reverse-transcribed in the later steps.

P2-10.1. The temperature cycles for the PCR are: 98 °C 3 min; 98 °C for 15 s, 56 °C for 30 s and 72 °C for 1 min, for 6 cycles; a final extension of 72 °C for 2 min.

P2-10.2. Visualize the PCR products on a 3% Low Range Ultra Agarose Gel.

Part 2-11. Reaction Clean-Up

A Wizard SV PCR Clean Up System (Promega) was used to remove the enzymes and excess primers. The manufacturer's instructions were followed with minor modifications at the elution step. Samples were eluted twice in 44 μL of sterile water.

*The reaction clean up step is critical to remove the polymerase enzyme which may fill the enzyme restriction sites after digestion. Moreover, it is also needed to remove the excess the 5' double biotin primers as they would bind the streptavidin beads with high efficiency and interfere with the streptavidin purification step.

Part 2-12. Enzyme Digestion

P2-12.1. Digest the PCR amplified and purified library material overnight at 65 °C with 20–100 U Taq1, 100 μg/mL BSA and 1× NEB 4 Buffer in a final volume of 100 μL.

P2-12.2. Use a top layer of sterile mineral oil to prevent evaporation and maintain the enzyme concentration steady.

Part 2-13. Streptavidin Purification

The GW and LA-COBRA libraries are biotin tagged at the amplification step. After *Taq1* digest, there are three main fragment types in the libraries; uncut biotinylated fragments (no internal *Taq1* site), the cut fragments containing 5' double biotin and the other part of the cut fragments which are non-biotinylated. In this step, Dynabeads® M-280 Streptavidin beads, which are ideal for purification of biotinylated molecules, were used to capture the biotinylated fragments, hence enrich for the non-biotinylated fragments.

P2-13.1. Resuspend the Dynabeads to obtain a homogenous suspension by shaking the vial.

P2-13.2. Transfer 100 μL of the beads into non-sticky microfuge tubes for each sample.

P2-13.3. Place the aliquots on a magnetic rack for 2 min to ensure all the beads are collected on the tube wall and remove the supernatants.

P2-13.4. Wash the beads twice in 100 μL 2× Binding and washing (B&W) Buffer (2×) with addition of 0.2% Tween 20 (10 mM Tris-HCl pH 7.5, 1 mM EDTA, 2.0 M NaCl and 0.2% Tween 20).

P2-13.5. Resuspend the beads in 100 μL 2× B&W Buffer with 0.2% Tween 20 and mix with an equal volume of Taq1 digested biotinylated library material.

P2-13.6. Gently mix the samples with a pipette and agitate at 300 rpm for 20 min at room temperature.

P2-13.7. Separate the beads coated with biotinylated library fragments using a magnet for 2 min.

P2-13.8. Remove the supernatant containing enriched non-biotinylated fragments and transfer into another tube.

The streptavidin beads can be uncoated from the biotinylated fragments with few additional steps. Although this is not a necessary step for GW and LA-COBRA library preparations, it is a good practice to visualize the discarded fragment on an agarose gel.

P2-13.9. Wash the beads twice with 1× B&W Buffer with addition of 0.1% Tween 20 (5 mM Tris-HCl pH 7.5, 0.5 mM EDTA, 1.0 M NaCl and 0.1% Tween 20).

P2-13.10. Resuspend the beads in 1× B&W Buffer containing 30 mM d-biotin and incubate at room temperature with gently mixing for 15 min

P2-13.11. Heat treat the samples are heated to 80 °C for 15 min.

P2-13.12. Place the samples on a magnet for 2 min and aspirate the supernatant containing the biotinylated fragments as soon as the magnetic beads were cleared from solution.

Part 2-14. Adapter-1 Ligation

Following digestion, the cut ends of DNA fragments are ligated to Adapter-1. This adapter has a 5'-CG-3' overhang and is not phosphorylated to reduce Adapter-1 to Adapter-1 ligation. The LA-COBRA Adapter-1 sequence incorporated an addition of P5 primer region that allows priming and reverse transcription on the T7 RNA polymerase-generated RNA transcripts.

P2-14.1. Prepare the ligation reaction with 1 µg of fragmented DNA with 2-fold molar excess of Adapter-1 in a 50 µL reaction containing 2000 U T4 Ligase Enzyme and 25 µL Quick Ligation Buffer.

P2-14.2. Incubate the reaction mixture at room temperature for 20 min and cool on ice.

The two-fold molar excess is calculated considering the library fragments contain two ligatable ends with an acceptance that average library fragments are 200 bp.

There are **X** moles of ligatable ends in the sonicated material,

$$\mathbf{X} = 1\ \mu\text{g DNA} \times 1\ (\text{single end})/(650\ \text{Da/bp} \times 200\ \text{bp}) = 7.8 \times 10^{-6} = 7.8\ \text{pmol}$$

$$\text{Adapter-1/Fragment} = 2{:}1 \qquad 7.8\ \text{pmol} \times 10 = 15.6\ \text{pmol}$$

Therefore, 0.32 µL of the 50 µM Adapter-1 stock is used for 1 µg library material.

Part 2-15. Adapter-1 Clean up

Adapter dimerization creates significant noise in the sequencing results, therefore purification steps are extremely important to remove any excess adapters. Ampure XP Bead Purification system is utilized to remove the fragments that have a size less than 100 bp using TruSeq DNA sample preparation guide with minor modifications.

P2-15.1. Cool down the Ampure XP beads to room temperature.

P2-15.2. Combine 25 µL of well mixed beads with 35 µL PCR water and 100 µL Sample.

P2-15.3. Gently mix the mixture via pipetting and incubate at room temperature for 15 min.

P2-15.4. Place the samples on a magnetic rack until the beads are collected to the tube wall.

P2-15.5. Remove the supernatant containing the fragments less than 100 bp.

P2-15.6. Wash the beads with 200 µL of 80% ethanol twice while the tubes are on the magnetic rack.

P2-15.7. Air dry the residual ethanol and resuspend the beads in 50–100 µL low TE for 2 min.

P2-15.8. Capture the beads on a magnetic rack.

P2-15.9. Transfer the solution containing the library material above 100 bp into a clean eppendorf.

Part 2-16. Check Point PCR

To test the presence of adapters in library fragments, perform PCRs in 25 µL volume using GoTaq white (Promega) in the buffer supplied with the enzyme, with the addition of 0.1 µM final concentration of Illumina flow cell primers on a fraction of the library.

P2-16.1. The temperature cycles for the PCR are: 98 °C for 5 min; 98 °C for 15 s, 65 °C for 30 s and 72 °C for 1 min, 8 cycles; a final extension of 72 °C for 2 min.

P2-16.2. Visualize the amplification products by electrophoresis on 3% Low Range Ultra Agarose (Biorad).

After the confirmation that library fragments contain Adapter-1 and Adapter-2 on the ends, GW-COBRA libraries are amplified using PCR whereas LA-COBRA libraries are amplified using *in vitro* transcription followed by cDNA synthesis.

Part 2-17. GW-COBRA: PCR Amplification

P2-17.1. Adapter 1 and 2 ligated GW-COBRA library fragments are used as templates with a final concentration of 1 ng/µL in 25 µL using GoTaq white (Promega) enzyme and buffer mixture.

P2-17.2. The Illumina flow cell primers are used in the final concentration of 0.1 µM.

P2-17.3. The temperature cycles for the PCR are: 98 °C 3 min; 98 °C for 15 s, 65 °C for 30 s and 72 °C for 1 min, for 7 cycles; a final extension of 72 °C for 2 min.

Note: It is important to use minimum number of PCR cycles to reduce PCR amplification bias towards CpG poor regions.

Part 2-18. LA-COBRA: *In vitro* Transcription

The LA-COBRA library is *in vitro* transcribed to RNA using T7 RNA Polymerase.

P2-18.1. Prepare the reaction buffer using 600 ng of library with 2 µL of 10 mM of ATP, 2 µL of 10 mM of GTP, 2 µL of 10 mM of UTP and 2 µL of 10 mM of CTP with 2 µL of T7 RNA Polymerase Mix in a total volume of 20 µL.

P2-18.2. Incubate the reaction in 37 °C overnight.

P2-18.3. Clean up using RNeasy MinElute Clean up kit.

P2-18.4. Quantify RNA concentration using NanoDrop 1000 Spectrophotometer with RNA settings as well as Quant-iT RNA assay as per manufacturer's protocol.

Part 2-19. cDNA Synthesis

P2-19.1. Mix approximately 600 ng (8 µL) of LA-COBRA transcripts with 1 µL of 100 µM P5 primer at 70 °C for 10 min then cool on ice for 2 min.

P2-19.2. Add the mixture to the SuperscriptIII first-strand synthesis reaction mix containing a final concentrations of 1× First strand buffer, 10 mM DTT, 1 mM dNTP mix with 40 U RNasin RNase inhibitor with 400 U SuperscriptIII enzyme in a final volume of 20 µL (QuantiTect® Reverse Transcription Kit).

P2-19.3. Incubate the reaction at 42 °C for an hour.

P2-19.4. Add RNase H (5 U) and RNase A (0.5 µg) into the reaction and further incubate at 37 °C for 15 min.

P2-19.5. Mix the first-strand synthesis reaction with a final concentration of 1× NEBuffer 2, 667 nM P7 primer and 0.3 mM dNTP mix in a final volume of 169 µL.

P2-19.6. Denature the final reaction mixture at 96 °C for 3 min and cool the reaction at 37 °C for 5 min.

P2-19.7. Add 5 units of Klenow fragment of DNA polymerase1 (exo-minus) and incubate the reaction at 37 °C for an hour.

Part 2-20. Size Selection by Ampure XP Bead Purification

The size selection is necessary to remove any adapter dimers and excess primers for two main reasons: the excess nucleotides will contribute to the library concentration quantifications and they may with the sequencing step, as the short fragments will amplify efficiently and populate the sequencing reads.

Use the Ampure XP Bead purification on both GW-COBRA and LA-COBRA final library materials as described earlier in Adapter-1 Clean Up step.

Part 2-21. Final Quantifications

DNA concentration in PCR amplified GW-COBRA and linear amplified LA-COBRA libraries post size exclusion is initially quantified using 1 µL of the samples on a NanoDrop 1000 Spectrophotometer (Thermo Fisher Scientific, Carlsbad, CA, USA). Considering the libraries contain small amounts of DNA, for accurate measurement, Quant-iT Picogreen dsDNA Reagent was used and the DNA concentrations are calculated according to a standard curve made using the Kit's control sample. Finally, the size distribution is visualized using Agilent DNA 1000 Assay in 2100 Bioanalyzer (Agilent Technologies, Los Angles, CA, USA) using the manufacturer's protocol.

3.2. Supplementary Materials and Methods Cont'd

Simulation Data

For a reasonable simulation we based the model on estimates from empirical data. The distribution of COBRA-seq read count data is zero-inflated negative binomial; some CpG sites are fully unmethylated so no amount of additional sequencing will raise read counts above zero, while for the rest of the CpG sites, read counts are proportional to a combination of sequencing depth and methylation rate. To model this distribution, the negative binomial parameters, mu (μ) and size, were estimated from Joint-COBRA data for sites with counts. In addition, the relationship between Joint-COBRA sites with 0 counts and 450K array beta value was examined and the correlation was found to be negatively exponential in nature.

As beta values are bimodal (most are near 0 or 1) we used empirical data as a source of "true" beta values for the simulation. In total, 200,000 beta values were sampled from 450K array data of HCT116 and these were considered true population means for CpG sites. Mean population methylated read counts were modelled as beta × coverage per site and unmethylated read counts as (beta-1) × coverage per site. These per site read count population means (λ) were used to derive 200,000 random Poisson samples for the methylated (M) and unmethylated (U) fractions and beta was constructed as beta (estimated) = M/(M + U). The estimated betas were plotted against true beta. For the COBRA-seq like, M only simulation, the same set of 200,000 random true betas were used to derive random negative binomial read counts using the parameters estimated from the empirical COBRA-seq data. To represent the additional CpG site

coverage possible when sequencing only the methylated genome fraction, the read coverage was moderated by divided by the mean beta value. Next, some counts were converted to 0. A total of 15.75% of CpG sites shared between the 450K array and Joint-COBRA had 0 counts and we used this as the fraction of CpG sites which are unmethylated—so we would expect no COBRA-seq counts even with high coverage. So, 15.75% of the 200,000 simulated read counts were randomly converted to 0, with a chance weighted by $(\text{beta} + 0.02)^{-2}$. The addition of 0.02 was to moderate the chance of selection of beta values very near 0 and beyond the distribution peak at approximately 0.03.

3.3. Supplementary Results and Discussion

Simulation Data

For absolute DNA methylation estimation it is usual to quantitate the methylated (M) fraction and unmethylated (U) fraction of a biological sample and to divide the methylated fraction by the sum of both fractions (M/(M + U)), to derive the beta value—the proportion of M. With low coverage bisulfite sequencing, this estimate of beta is granular and inaccurate. For example, with a 5-fold coverage at a CpG site, it is only possible to estimate methylation in steps of 20% and often the sampling of methylated and unmethylated reads will highly diverge from the true rate. As coverage increases, precision, accuracy and trueness slowly converge from low to high. If the unmethylated fraction (U) is removed, as in COBRA-seq and other methylated DNA enrichment methods, we observe a count, with the count proportional to M and the library size. While the ability to estimate the true rate of absolute methylation is lost, the property of accuracy and precision increasing with coverage is retained and proportionally, far more reads (than with WGBS) contribute DNA methylation information. We wished to examine this trade-off between absolute methylation estimation (M + U scenario) and more reads contributing methylation information (M-only scenario).

As read coverage increases in the M + U scenario, accuracy in beta estimation (emergence of a diagonal trend between 0 and 1) appears early, with the precision (the decrease in the breath of the line) improving slowly. As beta is ratio-based, over- or under-sampling of the M or U fraction will yield this imprecision. In the M-only case, accuracy and precision are a function of the population methylation, with accuracy and precision decreasing as the methylation rate increases. Very high counts are likely to represent a population methylation rate of 0.8–1.0, however the converse statement cannot be made. High True betas are captured as a wide range of count values.

Collectively, the simulation suggests the M-only case has higher precision than the M + U case for hypomethylated CpG sites and considerably worse precision for hypermethylated sites. However, the increasing imprecision for the M-only case, a dependence of the variance on the mean, can be dealt with by using a variance stabilizing transformation (VST), or similar techniques like those for RNA-seq count data [54]. This suggests COBRA-seq is well suited for finding methylation differences between groups of replicates (where variance can be modelled).

Methylation beta and count estimates are analogous to the FPKM and count-based methods in RNA-seq. It is reasonable to use a RNA-seq count software such as EdgeR to examine differences in methylation between groups of samples. Increasing variance with count is recognized as a property of RNA-seq count data and software such as EdgeR, DESeq2 and Voom will model the distribution and

moderate the counts. Like RNA-seq count-based methods, COBRA-seq is best suited to inter-sample comparisons and not intra-sample comparisons.

© 2015 by the authors; licensee MDPI, Basel, Switzerland. This article is an open access article distributed under the terms and conditions of the Creative Commons Attribution license (http://creativecommons.org/licenses/by/4.0/).
